# Purified Vero Cell Rabies Vaccine (PVRV, Verorab^®^): A Systematic Review of Intradermal Use Between 1985 and 2019

**DOI:** 10.3390/tropicalmed5010040

**Published:** 2020-03-07

**Authors:** Thomas Moulenat, Céline Petit, Valérie Bosch Castells, Guy Houillon

**Affiliations:** 1 Université Claude Bernard Lyon 1, Institut des Sciences Pharmaceutiques et Biologiques (ISPB)—Faculté de Pharmacie de Lyon, 69008 Lyon, France; thomas.moulenat@etu.univ-lyon1.fr; 2 Sanofi Pasteur, 14 Espace Henry Vallée, 69007 Lyon, France; Celine.Petit@sanofi.com (C.P.); Valerie.BoschCastells@sanofi.com (V.B.C.)

**Keywords:** immunogenicity, intradermal, post-exposure prophylaxis, pre-exposure prophylaxis, rabies vaccine, seroconversion

## Abstract

The purified Vero cell rabies vaccine (PVRV; Verorab^®^, Sanofi Pasteur) has been used in rabies prevention since 1985. Evolving rabies vaccination trends, including shorter intradermal (ID) regimens with reduced volume, along with WHO recommendation for ID administration has driven recent ID PVRV regimen assessments. Thus, a consolidated review comparing immunogenicity of PVRV ID regimens during pre-exposure prophylaxis (PrEP) and post-exposure prophylaxis (PEP) is timely and beneficial in identifying gaps in current research. A search of seven databases for studies published from 1985 to November 2019 identified 35 studies. PrEP was assessed in 10 studies (n = 926) with 1–3-site, 1–3-visit regimens of up to 3-months duration. Seroconversion (rabies virus neutralizing antibodies [RVNA] ≥ 0.5 IU/mL) rates of 90–100% were reported within weeks, irrespective of regimen, with robust booster responses at 1 year (100% seroconversion rates by day 14 post-booster). However, data are lacking for the current WHO-recommended, 2-site, 1-week ID PrEP regimen. PEP was assessed in 25 studies (n = 2136) across regimens of 1-week to 90-day duration. All ID PEP regimens assessed induced ≥ 99% seroconversion rates (except in HIV participants) by day 14–28. This review confirms ID PVRV suitability for rabies prophylaxis and highlights the heterogeneity of use in the field.

## 1. Introduction

Rabies, a zoonotic, acute progressive encephalomyelitis caused by rabies virus and other *Lyssavirus* species in the family *Rhabdoviridae*, is almost always fatal once clinical symptoms manifest [1]. It is considered a neglected disease that predominantly affects poor populations in rural locations [2,3]. The virus is usually transmitted through the saliva of infected animals following bites or scratches; dogs are responsible for most human rabies cases in rabies-endemic regions [4]. Globally there are an estimated 59,000 rabies-related deaths; the vast majority (over 95%) occurring in Asia and Africa [4,5]. Children are particularly at risk as they are more likely to receive severe bites on the face or neck or may not report apparently minor bites or scratches [4,6]. Approximately 40% of cases are in those aged <15 years. Travelers to rabies-endemic areas are also at risk of rabies, and imported cases continue to be reported periodically in otherwise rabies-free countries [7,8].

The first successful rabies prophylaxis, used on a patient with severe multiple bites from a rabid dog, was developed by Louis Pasteur and Emile Roux in 1885. Prophylaxis involved a subcutaneous injection with homogenates of rabies-infected rabbit nerve tissue that had been air-dried for 14/15 days, followed by 13 additional injections in 10 days with homogenates air-dried for gradually shorter periods and containing increasingly more virulent virus particles [9,10,11]. The method was successfully used for over half a century.

Rabies vaccines have subsequently undergone significant development to ensure consistency of virus inactivation, improve safety, and attempt to meet global demand. Purified cell culture and embryonated egg-based rabies vaccines (CCEEVs) have been available since the early 1960s. The World Health Organization (WHO) strongly recommended the discontinuation of nerve tissue vaccines production in 1984 because of the concerns with increased risk of severe adverse reactions and their lower immunogenicity compared with other available rabies vaccines [4]. However, a few countries with high rabies risk in Asia and Latin America continue to rely on animal nerve-tissue derived rabies vaccines for post-exposure prophylaxis [12].

Subsequent development of the human diploid cell vaccine (HDCV) and licensing in the mid- 1970s provided an alternative to CCEEVs with even fewer adverse effects [10]. Purified Vero cell rabies vaccines (PVRVs) were developed in the early 1980s and subsequently licensed in the mid- 1980s. One advantage of these vaccines is that they can easily be upscaled, if necessary, to meet industrial fermentation requirements, unlike HDCVs [10].

Rabies vaccines can be given both pre- and post-exposure to virus [4], unlike most other vaccine- preventable diseases. Pre-exposure prophylaxis (PrEP) rabies vaccination is recommended for travelers at risk of exposure, those in high-risk occupations, as well as children residing in or visiting, rabies-endemic areas [13]. Post-exposure prophylaxis (PEP) includes timely appropriate wound management and vaccination. Administration of rabies immune globulin (RIG) may additionally be required depending on both the severity of exposure and the individual’s rabies vaccination history. Rabies prophylaxis is considered 100% effective when provided in a timely and correct manner [1].

Rabies vaccines have traditionally been administered intramuscularly (IM); this was the standard mode of vaccine administration until 1991, when the WHO also recommended intradermal (ID) administration of modern rabies vaccines as a suitable alternative route [14]. ID administration has similar immunogenicity and safety to IM administration but requires less vaccine volume, and therefore preserves the limited stock available, which is a real concern in highly endemic Asian countries, as well as being economical and more affordable in resource limited countries [3]. Progressively shorter ID regimens requiring fewer injections and clinic visits (thus improving compliance) have been developed over the years without compromising efficacy [14,15]. Indeed, one dose of PVRV (ID or IM) may be sufficient PrEP as it can prime for an anamnestic antibody response to booster vaccination 1 year later [16]. Although ID administration requires specific experience to ensure correct vaccine administration technique, mainly to avoid accidental subcutaneous injection [14], arguably the skills are not difficult to acquire and are easily maintained as long as ID administration is done routinely.

PVRV (Verorab^®^, Sanofi Pasteur), has been available since 1985 for rabies PrEP and PEP [17]; initially approved for IM administration and subsequently for ID administration in 1996. Data on the first 20 years of clinical experience with the vaccine were reviewed previously [18]. However, there has been a gradual progression toward ID regimens (mainly in Asian countries) with PEP whereas ID administration in PrEP is still rare in both endemic and non-endemic countries. A detailed review summarizing the immunogenicity of ID administration of PVRV during rabies prophylaxis including recent studies is thus warranted and timely. This comprehensive review is intended to help consolidate the available PVRV ID immunogenicity data and identify possible gaps in the research. 

## 2. Materials and Methods

A literature search was executed across seven databases, Embase, Medline, Chemical Abstracts, Biosis, TrialTrove, ClinicalTrials.gov, and EudraCT to identify relevant studies published from 1985 to 21 November 2019 for inclusion in this review. The following keyword combination was used as appropriate across the articles databases: rabies vaccine OR (rabies* AND (vaccin* OR immuniz* OR pre exposure prophylaxis OR post exposure prophylaxis)) AND Verorab OR other Verorab tradenames OR Vero cell line OR Vero* OR pvrv* AND intradermal drug administration OR intradermal* OR intracutaneous* OR ID (within 2 words of vaccin* OR injection* OR syringe*). For the clinical trials databases, the following keyword combination was used as appropriate: Verorab OR PVRV OR ’purified inactivated Vero rabies vaccine’ OR ’purified Vero rabies vaccine’ AND intradermal OR ID.

Initially, the titles and abstracts (where available) of citations identified were appraised for relevance by T.M. (first author). The full text of potentially suitable studies was obtained and reviewed to identify relevant data. In addition, bibliographies of relevant review articles identified were searched for additional studies not captured by the database searches. Articles were chosen for inclusion in line with the PICOS approach [19]. There were no restrictions applied on the type of patient/participant. All prospective studies assessing ID PVRV during PrEP or PEP or following booster or simulated PEP that were available in English and reported primary immunogenicity data (i.e., rabies virus neutralizing antibodies [RVNA] titers, geometric mean titers [GMTs], seroconversion rates) were included. Data identified were extracted using MS Excel spread sheets/word tables. Where possible, RVNA titers/GMTs or seroconversion rates were summarized together, where the same assay method was used (typically the rapid fluorescent focus inhibition test [RFFIT] was used across the different studies), or presented individually for studies that used other assay methods where appropriate.

## 3. Results

**Figure 1:**
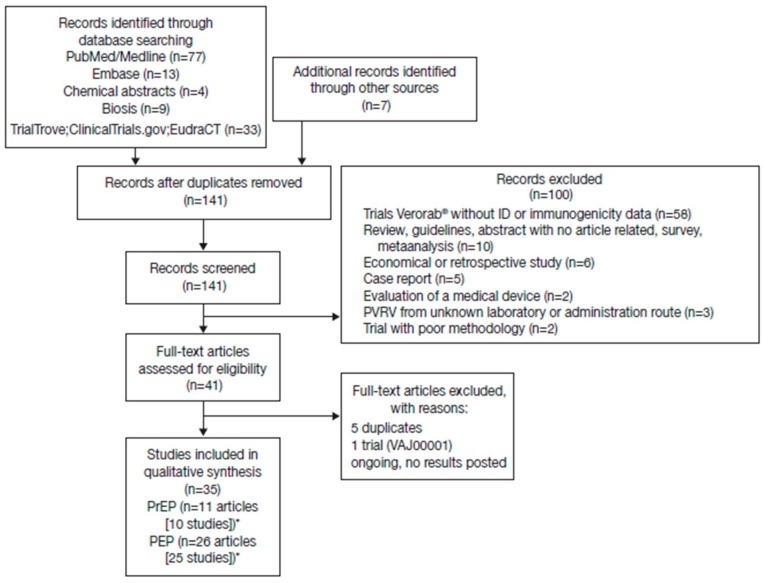
Studies identified through electronic database searches and other sources. *Some studies reported both pre-exposure prophylaxis (PrEP) and post-exposure prophylaxis (PEP) data.

A total of 144 potentially relevant citations were identified from the databases searched and other sources, of which 35 met the inclusion criteria (Figure 1). There was considerable heterogeneity across studies in terms of study designs, regimens, number of vaccination sites, vaccine potency, population assessed, and time series reported, precluding a meaningful pooled/meta-analysis of the RVNA titers. The intention here is to display the available data to allow the reader to assess general trends within selected groupings. Seroconversion was defined as a titer ≥ 0.5 IU/mL in all studies.

### 3.1. Immunogenicity of PrEP Regimens

There were 11 citations identified that reported immunogenicity data following ID PVRV for PrEP [16,20,21,22,23,24,25,26,27,28,29]; of these, 6 were undertaken in Thailand [20,23,25,28,29,30]. The other studies were undertaken in Vietnam [24,27] (both these Vietnamese studies reported data on the same cohort of children), Brazil [22], and The Netherlands [16,21]. The study populations included: children aged <1 year [24,27]; children aged 12–16 months [29]; children aged 5–12 years [23]; children and adults [25] or where age was not specified (assumed to be children and adults) [26]; and adults [16,20,21,22,28]. Details of the studies and immunogenicity data identified are summarized in Table S1 (Supplementary Materials).

#### 3.1.1. Immunogenicity Up to 1 Year

Since there is generally less urgency to ensure a rapid increase in RVNA titers with PrEP, there is less imperative to report/assess immunogenicity during the first few days or months after initiation or completion of vaccination, as was the case in most studies. However, some studies did report data for one or two selected time points through the first year. The potency of the PVRV used for PrEP varied from 2.5 to 8.7 IU/0.5 mL across studies.

**Figure 2:**
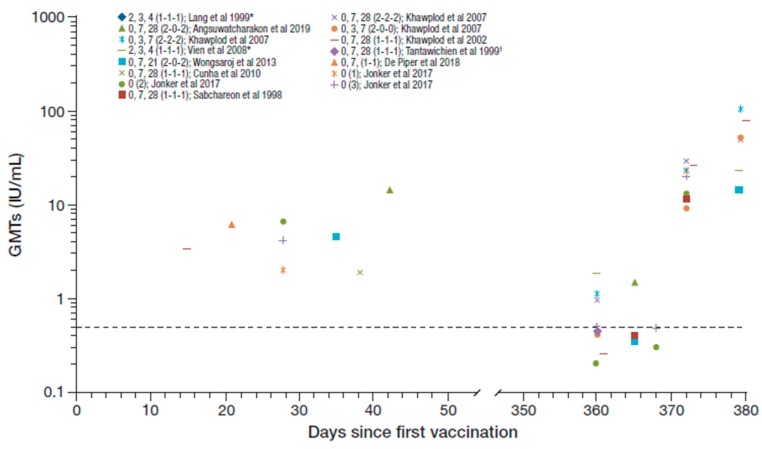
Summary of PrEP immunogenicity data from studies with purified Vero cell rabies vaccine (PVRV) intradermal (ID) identified irrespective of regimen [regimen summarized as day of vaccination (number of sites per vaccination day) in legend key]. Data shown to the right are post-booster geometric mean titers (GMTs). *The Lang et al. and Vien et al. studies are the same cohort, and regimen shows age at vaccination (i.e., at 2, 3, and 4 month of age). ^†^GMT reported as 0.4 and 0.43 in two groups (average 0.42 used here). Dotted line marks the 0.5 IU/ml rabies virus neutralizing antibodies (RVNA) titer.

Seroconversion rates from day 14 to 56 after the first vaccination and at 1 year across the studies identified were 90–100% and 20–96%, respectively [16,21,22,23,25,27,28,29]. The RVNA GMTs reported across the studies irrespective of PrEP regimen (n = 926), including following booster vaccination after one year, are summarized in Figure 2 [16,20,21,22,23,24,25,26,27,28,29]. The GMT range across studies from day 14 to 56 after the first vaccination was 1.90–14.35 IU/mL decreasing to ≤1.9 IU/mL at 1 year [16,20,21,22,23,26,29].

Five studies (n = 275) assessed immunogenicity data for 1- or 2-site, 3-visit, PrEP vaccination regimens (over a period up to 28 days, including one with a 1-week shortened regimen [25]) [22,23,25,26,28]. The previous WHO-recommended ID PrEP regimen was one injection given on each of days 0, 7, and 21 or 28 [13]. Seroconversion rates reported from day 14 to 56 after the first vaccination were reported as 96.9% and 98.9% in two of these studies [22,23], and GMTs reported ranged from 1.90 IU/mL to 4.7 IU/mL [22,23,26]. Immunogenicity data were reported at 1 year in four of the studies [23,25,26,28]; usually prior to a booster being given. Seroconversion rates reported at 1 year ranged from 38% to 94% and GMTs ranged from 0.26 IU/mL to 1.12 IU/mL.

Other PrEP regimens assessed included: 1-site, 3-month regimen (at age 23, 4 months) concomitant with DTP-IPV in children (n = 116) [24]; 2-site, 4-week regimen (0, 7, 28 [2-0-2]) with the first doses administered concomitantly with the chimeric live-attenuated Japanese encephalitis (JE) Vaccine (IMOJEV^®^) (n = 32) [29]; 1-site, 1-week regimen (0, 7 [1-1]) (n = 430) [21]; 2-site 3-week regimen (0, 7, 21 [2-0-2]) (n = 39) [20]; and 1-, 2- or 3-site single visit regimen (n = 10, 5, and 5, respectively) [16]. The three doses administered at monthly intervals concomitantly with DTP-IPV achieved a 100% seroconversion rate 15 weeks after the first dose and the reported GMT was 12.0 IU/mL [24]. There was no evidence of any interference between DTP-IPV and PVRV. The 2-site 4 weeks (0, 7, 28 [2-0-2]) regimen with the first doses administered concomitantly with the JE vaccine in children aged 12–24 months achieved a 100% seroconversion rate at day 42 which decreased to 92% at one year, with reported GMTs of 14.35 IU/mL and 1.5 IU/mL, respectively [29]. There was also no evidence of any interference between PVRV and the JE vaccine. Of note, the GMTs reported in studies with children (aged up to 12 years) [23,24,29] appear generally higher than those in adults only.

All participants who received the 2-site 3-week (0, 7, 21 [2-0-2]) regimen had seroconverted at day 35, but the seroconversion rate was not reported at year 1 for this study [20]. The corresponding 

GMTs achieved were 4.51 IU/mL at day 35 decreasing to 0.35 IU/mL at 1 year. The 1-site 1-week (0,7 [1-1]) regimen achieved a 99% seroconversion rate after day 21, and a GMT of 7.59 IU/mL [21]. The 1-, 2-, or 3-site single-visit regimens achieved seroconversion rates of 90%, 100%, and 100% at day 28, respectively, with reported GMTs of 2.0, 6.7, and 4.2 IU/mL. After one year, only 20%, 20%, and 40% remained seroconverted, respectively, and the GMTs in the groups decreased to below LLOQ, 0.2 and 0.5 IU/mL [16]. In another study, which included a 2-site single visit regimen, the seroconversion rate was 38.5% at 1 year with a corresponding GMT of 0.41 IU/mL [25].

#### 3.1.2. Booster Responses

Booster immunogenicity data were available for four studies; priming included 2-site single visit, 1- or 2-site 4-week (0, 7, 28 [2-2-2 or 1-1-1]) regimens and a 2-site 3-week (0, 7, 21 [2-0-2]) regimen [20,23,25,26]. These studies assessed the effect of boosting after 1 year with 1-site [23] or 4-site [26] administration at a single visit, or 1-site 3-day (0, 3 [1-1]) regimen [20,25]. All studies reported 100% seroconversion from day 7 to 14 post-booster, and GMTs ranged from 9.15 to 105.08 IU/mL [20,23,25,26].

Sabchareon et al. also reported RVNA persistence over the long-term in children post-booster; GMTs decreased from 11.8 IU/mL at day 7 to 1.6 IU/mL at day 730 post-booster with seroconversion maintained in 88.3% [23]. These long-term persistence data are reassuring considering the high rate of repeat exposure in children in Thailand (where the study was undertaken) [31]. Vien et al. reported RVNA persistence up to 5 years following booster in children who received ID PVRV concomitant with DTP-IPV one year earlier [27]; post-booster GMT decreased from about 23 IU/mL at day 14 to about 0.6 IU/mL at year 5 with seroconversion maintained in about 54%. Repeat booster at year 5 achieved 100% seroconversion at day 14 post-repeat booster and the GMT achieved was approximately 9.5 IU/mL.

Khawplod et al. assessed a 1-site 3-day (0, 3 [1-1]) booster regimen following priming with a 2- site single-visit ID PVRV one year earlier [25]; a robust immune response was observed following booster (all participants seroconverted and GMTs at day 7 and 14 post-booster were 9.15 and 51.96 IU/mL, respectively). Similarly, Jonker et al. 2017 [16] reported immunogenicity of IM (0.5mL IM, 3 days apart) booster 1 year after priming vaccination with 1, 2, or 3-site single-visit ID PVRV. All participants seroconverted and GMTs reported at day 7 post-booster were 22.6, 13.0, and 20.1 IU/mL in the 1-, 2-, and 3-site priming dose groups, respectively. These results taken together suggest that even single-visit regimens provide sufficient priming for subsequent ID or IM booster vaccination. 

Overall, the post-booster GMTs achieved across studies are suggestive of an amnestic response though there are limited immunogenicity data in the first few weeks following primary vaccination to allow for a more definitive comparison (Figure 2). Nonetheless, the available data are suggestive that even as single dose would provide adequate priming for subsequent booster response 1 year later [16].

### 3.2. Immunogenicity of PEP Regimens

There were 26 citations identified that reported immunogenicity data following ID PVRV as part of PEP [25,26,28,31,32,33,34,35,36,37,38,39,40,41,42,43,44,45,46,47,48,49,50,51,52,53]; of these 14 studies were undertaken in Thailand [25,28,30,32,33,34,37,39,42,44,46,47,48,51], and 5 in India [35,36,38,43,45]. Three studies were undertaken in The Philippines [40,50,52], and one included long-term follow up to year 5 [31,50]. The remainder were undertaken in Lithuania, the UK, and Cambodia [41,49,53]. Table S2 (Supplementary Materials) summarizes the details of the studies identified.

No studies were undertaken specifically in children alone; 13 studies included children and adults [25,33,34,38,39,40,45,46,47,50,51,52,53] or did not specify the age group [42], and the rest were in adults only. These studies were mainly undertaken in patients/participants without prior rabies vaccine experience (prior rabies vaccine experience was usually an exclusion criterion in these studies, except in three studies [28,32,33]). Unlike PrEP, there is urgency with PEP to ensure a rapid increase in RVNA titers. As such, most of these studies reported immunogenicity within the first 28 days (at day 7 and 14 in particular) after the initial vaccination. However, several studies also reported immunogenicity data at day 90 and at 1 year after initiation of vaccination. Figure S1 summarizes the available immunogenicity data (n = 2136) across all studies with available data. The potency of the PVRV used for PEP varied from 2.5 to 11.6 IU/0.5 mL across studies.

#### 3.2.1. Immunogenicity Up to 1 Year

Ten studies assessed simulated PEP in otherwise healthy participants with no prior rabies vaccination history [25,26,36,38,41,43,44,48,49,53]; these included one study where rabies vaccination history was not stated, but participants had no detectable RVNA at baseline [36], and one study in hemodialysis patients with no previous history of rabies vaccination [44]. The ID PVRV PEP vaccination schedule assessed varied considerably across studies (Table 1).

**Figure 3:**
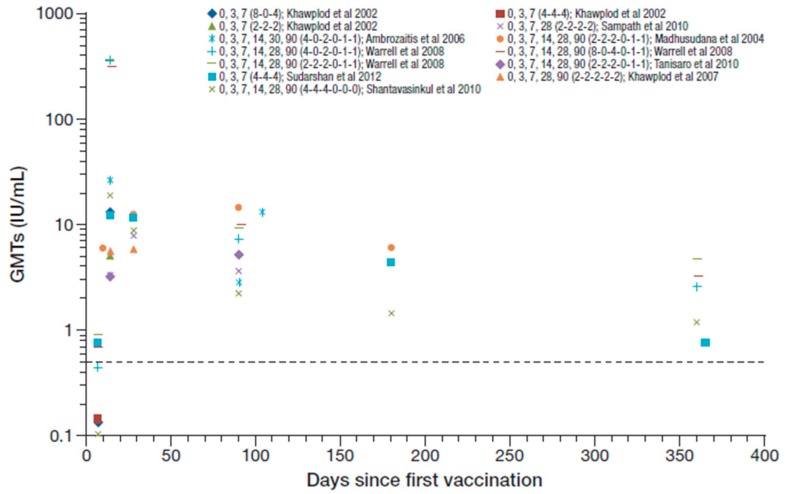
Summary of PEP immunogenicity data from studies with PVRV ID in otherwise healthy participants [regimen summarized as day of vaccination (number of sites per vaccination day) in legend key]. Dotted line marks the 0.5 IU/mL RVNA titer.

All participants in these studies were seroconverted by day 14 to 28 after the first vaccination, with reported GMTs ranging from 3.2 to 26.1 IU/mL [25,26,36,38,41,43,44,48]. Figure 3 summarizes the RVNA GMTs reported across these studies (Cantaert et al. reported median titers and are not included [53]). Warrell et al. 2008 reported GMTs ranging from 308–364 IU/mL at day 14 [49], which seem to be outlying data values compared to other studies and are not included in the ranges reported overall. The reasons for the unusual high levels in the later study were not described. One year after PEP, seroconversion was maintained in 63–100% in three studies that reported data at that time point [36,48] (including Warrell et al. 2008 [49]), and GMTs ranged from 0.75–4.6 IU/mL. Hemodialysis did not impair, or had only a minor effect on, RVNA responses [44].

Eleven studies assessed PEP that included rabies immune globulin (equine rabies immune globulin [ERIG] or human rabies immune globulin [HRIG]) [35,37,40,42,45,46,47,48,50,52,53], excluding one study in HIV patients reported in greater detail below [51]. The ID PVRV PEP vaccination regimens concomitant with RIG are also summarized in Table 1 along with the type of patient/participants assessed. One study reported combined immunogenicity data for those with, or without, concomitant HRIG [47].

**Figure 4:**
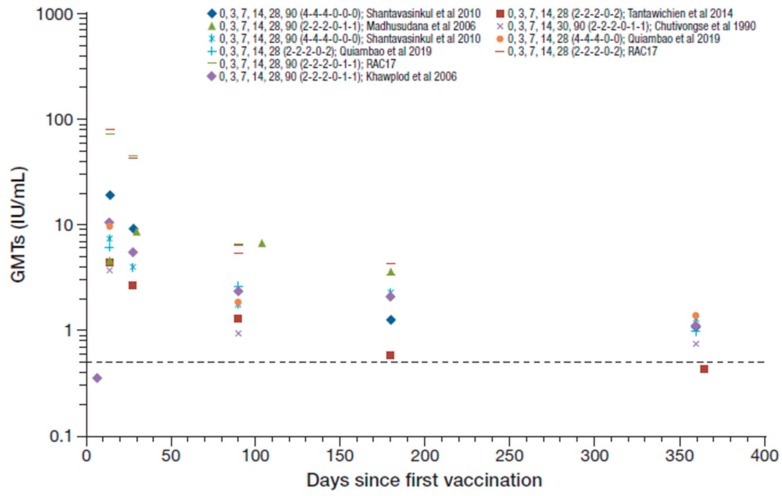
Summary of PEP immunogenicity data from studies with PVRV ID and concomitant rabies immune globulin (RIG) administration [regimen summarized as day of vaccination (number of sites per vaccination day) in legend key]. Dotted line marks the 0.5 IU/mL RVNA titer.

The studies that assessed regimens that included RIG reported 99–100% seroconversion rates from day 14 to 28 after the first vaccination (except for one study that reported 100% seroconversion at day 14 that decreased to 90% at day 28 [37]), with GMTs ranging from 2.7 to 19.7 IU/mL [42,45,46,48,50]. Figure 4 summarizes the RVNA GMTs reported across the studies that included concomitant RIG. The RAC17 study reported GMTs for two PVRV ID regimens (0,3,7,14,28,90 [2-2-2-0-1-1] and 0,3,7,14,28,90 [2-2-2-0-2-0]) of 80.8 and 72.9 IU/mL at day 14, respectively, and 43.8 and 45.4 IU/mL at day 28 [52], which are outlying data compared to other studies and were therefore not included in the ranges reported overall. Seroconversion rates were maintained at 50–100% at 1 year, with GMTs decreasing to 0.42–1.37 IU/mL [37,42,46,48,50]. These results were, in general, slightly lower than those reported without RIG use in simulated PEP in otherwise healthy participants. 

There were four studies in low risk patients including WHO category I or II rabies virus exposure (see Table 1 for regimens assessed) [34,39,40,50]. All participants seroconverted by day 14 to 28 after the first vaccination (except in one study where seroconversion rate was 86% on day 14 increasing to 100% on day 28 [39]) [34,40,50], with GMTs ranging from 5.07 to 15.5 IU/mL [34,39,50].

Tantawichien et al. assessed the immunogenicity of ID PVRV PEP (0, 3, 7, 30, 90 [4-4-4-0-2-2] regimen) in patients with HIV (n = 10), of whom nine had WHO category III rabies virus exposure and received concomitant HRIG [51]. Some of these patients had T lymphocyte counts <200/µL, but only four were treated with antiretroviral drugs. Seroconversion was achieved in 70%, 78%, and 71% at day 14, day 30, and day 90, respectively, with GMTs ranging from <0.04 to 10.37 IU/mL during that time. This study showed that HIV-infected patients generally have a poor response to PEP, despite the double dose used in these patients compared to that used in most other studies. Three of seven patients with CD4^+^ T lymphocyte counts of ≤200/µL had RVNA titers <0.5 IU/mL on day 14 or undetectable antibodies; one patient had undetectable RVNAs throughout most of the study. The other three patients with CD4+ T lymphocyte counts of >200/µL all achieved seroconversion at day 14.

#### 3.2.2. Persistence of PEP Immunogenicity

One randomized controlled study reported persistence of PEP immunogenicity annually from year 1 to 5 post-vaccination [31,50]. Seroconversion rates remained >95% in the group that received the shortened 1-week 4-site ID (4-4-4-0-0) regimen with PVRV. GMTs declined from 2.96 IU/mL at year 1 to 2.22 IU/mL at year 5. Concomitant ERIG appeared to attenuate RVNA titers though this effect varied by PEP vaccination regimen. Seroconversion rates remained relatively stable, between 80% and 90% in the group that received a shortened 1-week 4-site ID (4-4-4-0-0) regimen with ERIG (GMTs declined from 1.37 IU/mL to 1.05 IU/mL), but decreased from 80% at year 1 to 64% at year 5 in the group that received an updated 2-site Thai Red Cross (0, 3, 7, 14, 28 [2-2-2-0-2]) with ERIG (GMTs declined from 0.97 IU/mL to 0.76 IU/mL).

#### 3.2.3. Booster Response

Three studies assessed healthy subjects with prior PrEP or PEP 1 year (0, 3 [4-0] regimen), 12–16 months (0, 3 [1-1] regimen) [28,32], and 1–10 years (4-site single visit) earlier [33]. In two studies [28,32], all participants seroconverted by day 14 after the first booster vaccination, and the GMTs reported were 76.4 and 10.78 IU/mL, respectively; no data were reported during the time points considered in the other study. These studies in general reported GMTs that were similar to those for simulated PEP in otherwise healthy participants with no prior rabies vaccine history.

Four studies assessed PEP followed by simulated (booster) PEP after 1 year [36,37,40] or 5 years [31]. Of these, two studies assessed simulated PEP in otherwise healthy participants after 1 year [36,37]. In the first study, participants with RVNA levels that fell to <0.5 IU per mL at day 365 received a 4-site booster at day 436 [36]. All who received booster seroconverted by day 7, and GMTs at day 7 and day 14 post-booster were 3.60 IU/mL (range 2.5–5.7]) and 8.62 IU/mL (range 6.5–10.6), respectively. The GMT profile in the first few days in this group appears similar to that seen with simulated PEP in those with no prior rabies vaccination history ([ 3). In the second study, 100% seroconversion rate was reported at day 14 after initiation of booster (0, 3 [1-1] regimen) and the GMT was 15.12 IU/mL [37]. The other two studies assessed simulated PEP after at least 1 year (mean 419 days) and at 5 years following prophylaxis for category I/II [40] or II/III [31] exposure, respectively. In category I/II patients (1-site booster), 100% seroconversion rate was achieved by day 14 post- booster and the GMT was >10 IU/mL [40]. The category II/III patients (4-site booster) achieved a robust anamnestic response (100% seroconversion rates with GMTs ranging from 137 to 193 IU/mL at day 11 post-booster). However, the relative attenuation of RVNA titers with ERIG appears to persist with the booster [31].

## 4. Discussion

Although the WHO has provided recommendations for PrEP and PEP regimens, national guidelines differ between countries that have contributed to the assessment of numerous regimens over the years [54]. In this regard, it is reassuring that the immunogenicity of ID PVRV has been extensively assessed using numerous PrEP, PEP, and simulated PEP regimens. All ID PVRV PrEP regimens appear to be immunogenic, with 90–100% seroconversion rates reported at various time points within the first few days/weeks after the last vaccination dose, even with a single ID dose. The 1- or 2-site 3-week regimens (previously recommended or resembling those previously recommended by WHO) provides sufficient priming for a robust booster response. Data on alternate ID PVRV PrEP regimens are generally limited; specifically, data are lacking for PVRV administered according to the current WHO recommended ID PrEP, 2-site, 1-week (0, 7 [2-2]) regimen [55].

In addition, all ID PEP regimens assessed were highly immunogenic; seroconversion was achieved in nearly all subjects (≥ 99%) assessed between days 14 and 28 after initiation of vaccination, across all studies (except those with HIV-positive patients). The use of RIG appears to attenuate RVNA GMTs though this does not appear to be clinically meaningful. Immunogenicity data for PEP with ID PVRV administered according to the current WHO recommended ID regimen (2-site, 1-week (0,3, 7 [2-2-2] regimen [Institute Pasteur Cambodia, IPC regimen]) are limited [55]; only one small study with simulated PEP (n = 22) assessed the current recommended regimen [26]. However, sufficient clinical data has accumulated over the years with other WHO-approved rabies vaccines to support the recommended abridged regimens [14,15,56], and that these vaccines products could be used interchangeably during the course of PrEP or PEP in practice [55].

RVNA titers declined over time with both PrEP and PEP regimens in all studies. In the PrEP studies that reported immunogenicity at 1 year following initiation of immunization [16,20,23,25,26,29,57], only two studies reported that GMTs remained ≥0.5 IU per mL (0, 7, 28 [2-2-2] and 0, 3, 7 [2-2-2] regimens [25] and 0, 7, 28 [2-0-2] regimen [57]). Ideally, RVNA titers should remain ≥0.5 IU/mL following PrEP for the longest duration possible as this is usually the lower limit required to initiate booster immunization as “an additional precaution for those whose occupation puts them at continual or frequent risk of exposure” [4]. In contrast, most PEP studies that reported immunogenicity at 1 year following initiation of immunization showed that GMTs remained ≥ 0.5 IU per mL including regimens that included RIG [28,35,36,42,46,48,49,50] (the only exception was Tantawichien et al [37]). Seroconversion rates at 1 year were maintained at about 80–100% in all but two studies [36,37]. Of note, the shortened 1-week, 4-site ID PVRV (0, 3, 7, [4-4-4]) regimen was shown to achieve persistently higher RVNA titers than the updated 2-site Thai Red Cross (TRC) (0, 3, 7, 14, 28 [2-2-2-0-2]) regimen, and higher seroconversion rates up to five years after the first dose of primary immunization [31].

All ID PVRV PrEP regimens (including the 1-visit regimens) assessed provided sufficient priming for a robust booster response after 1 year. However, studies assessing booster responses after longer time periods since PrEP are lacking. One study reported that seroconversion persisted in 54% up to 5 years after booster, with GMT of about 0.6 IU/mL [27]; a robust immune response was observed with another booster at 5 years. In addition, simulated PEP (booster) up to 5 years after initial PEP immunization induced a robust anamnestic response [31]. The latter observation is reassuring considering the high number of repeat bites/potential exposure. Indeed, one dose may be sufficient to prime for rapid anamnestic antibody response to booster vaccination within 7 days, even among those who do not achieve or whose RVNA levels fell below the 0.5 IU/ml threshold [16]. Moreover, adequate booster responses can still be achieved after a long interval [58]. Thus, the WHO also now acknowledges that no further boosters are required following primary series of PrEP or PEP [55].

There was no evidence of any interference between ID PVRV PrEP and the JE vaccine or DTP- IPV in children [24,29]. As such, there is the potential to include ID PVRV PrEP into childhood immunization schedules in rabies-endemic countries. However, this may be unlikely in developing countries due to other competing priorities as well as the need for PEP upon future exposures to rabies virus [59]. A recent modelling study showed that PrEP as part of routine childhood immunization was unlikely an efficient use of resources, except where the incidence of rabies virus exposures was extremely high [60]. Nonetheless, the current WHO-recommended ID PrEP regimen requires four doses over two visits (0, 3, 7 [2-0-2] regimen) [55] rather than three doses over three visits as (0, 7, 21/28 [1-1-1] regimen) previously recommended [13]. Although this current regimen requires additional vaccine volume, it reduces the number of visits and associated logistics and therefore may have cost advantages over the previous regimen.

There are a number of limitations to this systematic review. There was considerable heterogeneity in PrEP and PEP regimens and potency of PVRV administered. Most studies assessed small sample sizes (n < 50), which may limit the generalizability of the observations to the wider population. In addition, studies undertaken in Africa were lacking. Data for PrEP were reported at selected time points that were generally inconsistent between studies. One of the advantages of this review is that the RFFIT was used across most studies to measure RVNA titers (in all but two studies [16,43]). Using the same method decreases the source of variability when comparing RVNA results since RVNA assays are complex and potential variations in performance characteristics may be observed [61].

This systematic review shows that although the RVNA threshold of ≥ 0.5 IU/mL achieved with ID PVRV PrEP may not be maintained through to 1 year after initiation of vaccination, nonetheless, the priming induced irrespective of regimen is sufficient for a robust RVNA response to PEP. In addition, ID PVRV PEP rapidly induces RVNA titers that exceed the 0.5 IU/mL threshold, irrespective of concomitant use of RIG across the numerous regimens assessed, which appears to persist above the threshold for at least five years in the majority of subjects. Although numerous PrEP and PEP regimens with ID PVRV have been assessed, data are generally lacking for current, recently updated, WHO-recommended ID regimens. In particular, the current recommended 2-site 1-week (2-0-2) PrEP regimen has not been assessed with ID PVRV; a study is currently being conducted to investigate this schedule (NCT03700242 [VAJ00001 study]). The diversity/heterogeneity/variability in regimen/potency/population demonstrated in this review underscores the continued need for studies to examine immunogenicity related to ID administration both across vaccine type and regimen in multiple populations.

## Figures and Tables

**Table 1:** PEP ID regimens assessed.

Regimen; Days (Number of Sites) [n]	Patients/Participants	Source
0, 3, 7 (2-2-2) [n = 21]	Simulated PEP (healthy participants)	[26]
0, 3, 7 (4-4-4) [n = 21]	Simulated PEP (healthy participants)	[26]
0, 3, 7 (4-4-4) [n = 40]	Simulated PEP (healthy participants)	[36]
0, 3, 7 (8-0-4) [n = 22]	Simulated PEP (healthy participants)	[26]
0, 3, 7, 14, 28 (2-2-2-0-2) [n = 63]	Simulated PEP (healthy participants)	[38]
0, 3, 7, 14, 28 (2-2-2-0-2) [n = 20]	Simulated PEP (healthy participants)	[53]
0, 3, 7, 14, 28, 90 (2-2-2-0-1-1) [n = 38]	Simulated PEP (healthy participants)	[43]
0, 3, 7, 14, 28, 90 (2-2-2-0-1-1) [n = 58]	Simulated PEP (healthy participants)	[49]
0, 3, 7, 14, 28, 90 (2-2-2-0-1-1) [n = 14]	Simulated PEP (hemodialysis patients)	[44]
0, 3, 7, 14, 28, 90 (2-2-2-0-2-2) [n = 10]	Simulated PEP (healthy participants)	[25]
0, 3, 7, 14, 28, 90 (4-0-2-0-1-1) [n = 55]	Simulated PEP (healthy participants)	[49]
0, 3, 7, 14, 30, 90 (4-0-2-0-1-1) [n = 87]	Simulated PEP (healthy participants)	[41]
0, 3, 7, 14, 28, 90 (8-0-4-0-1-1) [n = 58]	Simulated PEP (healthy participants)	[49]
0, 3, 7, 14, 28, 90 (4-4-4-0-0-0) [n = 45]	Simulated PEP (healthy participants)	[48]
0, 3, 7 (2-2-2) [n = 15]	Category I/II	[34]
0, 3, 7 (4-4-4) [n = 182]	Category I	[50]
0, 3, 7, 28 (1-1-1-1) [n = 14]	Category I/II	[39]
0, 3, 7, 28 (2-2-2-2) [n = 15]	Category I/II	[39]
0, 3, 7, 28 (4-4-4-4) [n = 14]	Category I/II	[39]
0, 3, 7, 14, 28, 90 (4-0-2-0-1-1) [n = 96]	Category I/II	[40]
0, 3, 7, 14, 28, 90 (8-0-4-0-1-1) [n = 96]	Category I/II	[40]
0, 3, 7 (4-4-4) [n = 44]	Category II/III/ with or without ERIG groups combined	[35]
0, 3, 7, 14, 28 (4-4-4-0-0) [n = 170]	Category III/concomitant ERIG	[50]
0, 3, 7, 14, 28, 90 (4-4-4-0-0-0) [n = 45]	Simulated PEP (healthy participants)/concomitant ERIG	[48]
0, 3, 7, 14, 28, 90 (4-4-4-0-0-0) [n = 41]	Category III/concomitant ERIG	[48]
0, 3, 7, 14, 28, 90 (2-2-2-0-1-1) [n = 99]	Category I/II/concomitant ERIG	[40]
0, 3, 7, 14, 28 (2-2-2-0-2) [n = 31]	Simulated PEP (healthy participants)/concomitant ERIG	[37]
0, 3, 7, 14, 28 (2-2-2-0-2) [n = 177]	Category III/concomitant ERIG	[50]
0, 3, 7, 14, 28 (2-2-2-0-2) [n = 116]	Category III/concomitant ERIG	[53]
0, 3, 7, 14, 28 (2-2-2-0-2) [n = 82]	Category I–III/concomitant ERIG	[52]
0, 3, 7, 14, 28, 90 (2-2-2-0-1-1) [n = 50]	Category III/concomitant ERIG	[45]
0, 3, 7, 14, 28, 90 (2-2-2-0-1-1) [n = 10]	High risk exposure/concomitant ERIG	[46]
0, 3, 7, 14, 28, 90 (2-2-2-0-1-1) [n = 48]	Category I–III/concomitant ERIG	[52]
0, 3, 7, 14, 28, 90 (2-2-2-0-1-1) [n = 105]	Category III/concomitant HRIG	[42]
0, 3, 7, 14, 28, 90 (2-2-2-0-1-1) [n = 59]	Category II/III/ concomitant HRIG (groups combined)	[47]
0, 3, 7, 14, 30, 90 (4-4-4-0-2-2) [n = 10]	Category III/ HIV patients/concomitant ERIG	[51]
0, 3 (4-0) [n = 20]	Prior PreP 1 year earlier/simulated PEP (booster)	[28]
0, 3 (4-0) [n = 10]	Prior PrEP or PEP 1–10 years earlier/simulatedPEP (booster)	[33]
0, 3 (1-1) [n = 35]	Prior PreP 12–16 month earlier/simulated PEP (booster)	[32]

## Supplementary Materials

The following are available online at https://www.mdpi.com/2414-6366/5/1/40/s1. Figure S1: Summary of post-exposure prophylaxis (PEP) immunogenicity data from all studies irrespective of regimen [regimen summarized as day of vaccination (number of sites per vaccination day) in legend key]. Table S1: Summary of publications on pre-exposure rabies prophylaxis (PrEP) with the purified Vero cell rabies vaccine (PVRV) identified through the systematic review of the literature up to 2019. Table S2: Summary of publications on post- exposure rabies prophylaxis (PEP) with the purified Vero cell rabies vaccine (PVRV) identified through the systematic review of the literature up to 2019.

## References

[1] Fooks A.R., Cliquet F., Finke S., Freuling C., Hemachudha T., Mani R.S., Muller T., Nadin-Davis S., Picard-Meyer E., Wilde H. (2017). Rabies. Nat. Rev. Dis. Prim..

[2] Wunner W.H., Briggs D.J. (2010). Rabies in the 21 century. PLoS Negl. Trop. Dis..

[3] Hampson K., Cleaveland S., Briggs D. (2011). Evaluation of cost-effective strategies for rabies post-exposure vaccination in low-income countries. PLoS Negl. Trop. Dis..

[4] World Health Organization (2018). Rabies vaccines: WHO position paper—April 2018. Wkly. Epidemiol. Rec..

[5] World Health Organization (2019). Rabies. https://www.who.int/news-room/fact-sheets/detail/rabies.

[6] World Health Organization (2018). Frequently Asked Questions about Rabies for the General Public. https://www.who.int/rabies/Rabies_General_Public_FAQs_21Sep2018.pdf?ua=1.

[7] Yamamoto S., Iwasaki C., Oono H., Ninomiya K., Matsumura T. (2008). The first imported case of rabies into Japan in 36 years: A forgotten life-threatening disease. J. Travel Med..

[8] Smith J., McElhinney L., Parsons G., Brink N., Doherty T., Agranoff D., Miranda M.E., Fooks A.R. (2003). Case report: Rapid ante-mortem diagnosis of a human case of rabies imported into the UK from the Philippines. J. Med. Virol..

[9] Hicks D.J., Fooks A.R., Johnson N. (2012). Developments in rabies vaccines. Clin. Exp. Immunol..

[10] Wu X., Smith T.G., Rupprecht C.E. (2011). From brain passage to cell adaptation: The road of human rabies vaccine development. Expert Rev. Vaccines.

[11] Pearce J.M. (2002). Louis Pasteur and rabies: A brief note. J. Neurol. Neurosurg. Psychiatry.

[12] World Health Organization (2015). Rabies. https://www.who.int/biologicals/vaccines/rabies/en/.

[13] World Health Organization (2013). WHO Expert Consultation on Rabies. Second report. WHO Technical Report Series No. 982..

[14] Gongal G., Sampath G. (2018). Introduction of intradermal rabies vaccination—A paradigm shift in improving post-exposure prophylaxis in Asia. Vaccine.

[15] Tarantola A., Tejiokem M.C., Briggs D.J. (2019). Evaluating new rabies post-exposure prophylaxis (PEP) regimens or vaccines. Vaccine.

[16] Jonker E.F.F., Visser L.G. (2017). Single visit rabies pre-exposure priming induces a robust anamnestic antibody response after simulated post-exposure vaccination: Results of a dose-finding study. J. Travel Med..

[17] Pichon S., Guinet-Morlot F., Minutello M., Donazzolo Y., Rouzier R., Chassard D., Fitoussi S., Hou V. (2013). A serum-free, purified vero cell rabies vaccine is safe and as immunogenic as the reference vaccine Verorab for pre-exposure use in healthy adults: Results from a randomized controlled phase-II trial. Vaccine.

[18] Toovey S. (2007). Preventing rabies with the Verorab vaccine: 1985-2005 Twenty years of clinical experience. Travel Med. Infect. Dis..

[19] Centre for Reviews and Dissemination (2006). Systematic Reviews: CRD’s Guidance for Undertaking Reviews in Health Care. https://www.york.ac.uk/media/crd/Systematic_Reviews.pdf.

[20] Wongsaroj P., Udomchaisakul P., Tepsumethanon S., Khawplod P., Tantawichien T. (2013). Rabies neutralizing antibody after 2 intradermal doses on days 0 and 21 for pre-exposure prophylaxis. Vaccine.

[21] De Pijper C.A., Boersma J., Terryn S., Van Gucht S., Goorhuis A., Grobusch M.P., Stijnis C. (2018). Rabies antibody response after two intradermal pre-exposure prophylaxis immunizations: An observational cohort study. Travel Med. Infect. Dis..

[22] Cunha R.S., Silva Ade C., Batista A.M., Chaves L.B., Barata R.B. (2010). Equivalence between pre-exposure schemes for human rabies and evaluation of the need for serological monitoring. Rev. Saude Publica.

[23] Sabchareon A., Chantavanich P., Pasuralertsakul S., Pojjaroen-Anant C., Prarinyanupharb V., Attanath P., Singhasivanon V., Buppodom W., Lang J. (1998). Persistence of antibodies in children after intradermal or intramuscular administration of preexposure primary and booster immunizations with purified Vero cell rabies vaccine. Pediatr. Infect. Dis. J..

[24] Lang J., Hoa D.Q., Gioi N.V., Vien N.C., Nguyen C.V., Rouyrre N., Forrat R. (1999). Immunogenicity and safety of low-dose intradermal rabies vaccination given during an Expanded Programme on immunization session in Viet Nam: Results of a comparative randomized trial. Trans. R. Soc. Trop. Med. Hyg..

[25] Khawplod P., Wilde H., Benjavongkulchai M., Sriaroon C., Chomchey P. (2007). Immunogenicity study of abbreviated rabies preexposure vaccination schedules. J. Travel Med..

[26] Khawplod P., Benjavongkulchai M., Limusanno S., Chareonwai S., Kaewchompoo W., Tantawichien T., Wilde H. (2002). Four-site intradermal postexposure boosters in previously rabies vaccinated subjects. J. Travel Med..

[27] Vien N.C., Feroldi E., Lang J. (2008). Long-term anti-rabies antibody persistence following intramuscular or low- dose intradermal vaccination of young Vietnamese children. Trans. R. Soc. Trop. Med. Hyg..

[28] Tantawichien T., Benjavongkulchai M., Limsuwan K., Khawplod P., Kaewchompoo W., Chomchey P., Sitprija V. (1999). Antibody response after a four-site intradermal booster vaccination with cell-culture rabies vaccine. Clin. Infect. Dis..

[29] Angsuwatcharakon P., Ratananpinit N., Yoksan S., Saengseesom W., Sriaksorn R., Raksahket N., Tantawichien T. (2019). Immunogenicity and safety of a double-dose, two-visit, pre-exposure rabies prophylaxis regimen versus a conventional regimen with Vero cell rabies and concomitant chimeric live-attenuated Japanese encephalitis vaccine administration. Vaccine.

[30] Khawplod P., Wilde H., Tepsumethanon S., Limusanno S., Tantawichien T., Chomchey P., Ayuthaya A.B., Wangroonsarb Y. (2002). Prospective immunogenicity study of multiple intradermal injections of rabies vaccine in an effort to obtain an early immune response without the use of immunoglobulin. Clin. Infect. Dis..

[31] Quiambao B.P., Ambas C., Diego S., Bosch Castells V., Korejwo J., Petit C., Rasuli A., Houillon G. (2019). A single-visit, 4-site intradermal (ID) rabies vaccination during simulated post-exposure induces a robust immune response 5 years after primary 1-week, 4-site ID post-exposure prophylaxis regimen in the Philippines. Vaccine.

[32] Kositprapa C., Limsuwun K., Wilde H., Jaijaroensup W., Saikasem A., Khawplod P., Kri-aksorn U., Supich C. (1997). Immune response to simulated postexposure rabies booster vaccinations in volunteers who received preexposure vaccinations. Clin. Infect. Dis..

[33] Tantawichien T., Tantawichien T., Supit C., Khawplod P., Sitprija V. (2001). Three-year experience with 4-site intradermal booster vaccination with rabies vaccine for postexposure prophylaxis. Clin. Infect. Dis..

[34] Phanuphak P., Khaoplod P., Benjavongkulchai M., Chutivongse S., Wilde H. (1990). What happens if intradermal injections of rabies vaccine are partially or entirely injected subcutaneously?. Bull. World Health Organ..

[35] Narayana A., Manoharan A., Narayan M.S., Kalappa S.M., Biligumba G., Haradanahalli R., Anand A.M. (2015). Comparison of safety and immunogenicity of 2 WHO prequalified rabies vaccines administered by one week, 4 site intra dermal regimen (4-4-4-0-0) in animal bite cases. Hum. Vaccines Immunother..

[36] Sudarshan M.K., Narayana D.H., Madhusudana S.N., Holla R., Ashwin B.Y., Gangaboraiah B., Ravish H.S. (2012). Evaluation of a one week intradermal regimen for rabies post-exposure prophylaxis: Results of a randomized, open label, active-controlled trial in healthy adult volunteers in India. Hum. Vaccines Immunother..

[37] Tantawichien T., Sibunruang S., Tantawichien T., Angsanakul J., Benjavongkulchai M., Limsuwan K., Udomchaisakul P., Khomvilai S., Sitprija V. (2014). Safety and immunogenicity of chromatographically purified Vero cell rabies vaccine for intradermal pre- and post-exposure rabies prophylaxis. Expert Rev. Vaccines.

[38] Sampath G., Madhusudana S.N., Sudarshan M.K., Ashwathnarayana D.H., Mahendra B.J., Ullas T.P., Mohan K., Madhusudhan S.K., Ravish H.S. (2010). Immunogenicity and safety study of Indirab: A Vero cell based chromatographically purified human rabies vaccine. Vaccine.

[39] Phanuphak P., Khawplod P., Sirivichayakul S., Siriprasomsub W., Ubol S., Thaweepathomwat M. (1987). Humoral and cell-mediated immune responses to various economical regimens of purified Vero cell rabies vaccine. Asian Pac. J. Allergy Immunol..

[40] Quiambao B.P., Gepanayao C., Bermal N., Ambas M.C., Dy-Tioco H., Crisostomo M., Dizon R. (2008). Rabies vaccination regimens using purified Vero cell rabies vaccine. APCRI J..

[41] Ambrozaitis A., Laiskonis A., Balciuniene L., Banzhoff A., Malerczyk C. (2006). Rabies post-exposure prophylaxis vaccination with purified chick embryo cell vaccine (PCECV) and purified Vero cell rabies vaccine (PVRV) in a four-site intradermal schedule (4-0-2-0-1-1): An immunogenic, cost-effective and practical regimen. Vaccine.

[42] Khawplod P., Wilde H., Sirikwin S., Benjawongkulchai M., Limusanno S., Jaijaroensab W., Chiraguna N., Supich C., Wangroongsarb Y., Sitprija V. (2006). Revision of the Thai Red Cross intradermal rabies post-exposure regimen by eliminating the 90-day booster injection. Vaccine.

[43] Madhusudana S.N., Sanjay T.V., Mahendra B.J., Suja M.S. (2004). Simulated post-exposure rabies vaccination with purified chick embryo cell vaccine using a modified Thai Red Cross regimen. Int. J. Infect. Dis..

[44] Tanisaro T., Tantawichien T., Tiranathanagul K., Susantitaphong P., Chirananthavat T., Praditpornsilpa K., Sitprija V., Eiam-Ong S. (2010). Neutralizing antibody response after intradermal rabies vaccination in hemodialysis patients. Vaccine.

[45] Madhusudana S.N., Sanjay T.V., Mahendra B.J., Sudarshan M.K., Narayana D.H., Giri A., Muhamuda K., Ravi V., Vakil H.B., Malerczyk C. (2006). Comparison of saftey and immunogenicity of purified chick embryo cell rabies vaccine (PCECV) and purified vero cell rabies vaccine (PVRV) using the Thai Red Cross intradermal regimen at a dose of 0.1 ML. Hum. Vaccines.

[46] Chutivongse S., Wilde H., Supich C., Baer G.M., Fishbein D.B. (1990). Postexposure prophylaxis for rabies with antiserum and intradermal vaccination. Lancet.

[47] Briggs D.J., Banzhoff A., Nicolay U., Sirikwin S., Dumavibhat B., Tongswas S., Wasi C. (2000). Antibody response of patients after postexposure rabies vaccination with small intradermal doses of purified chick embryo cell vaccine or purified Vero cell rabies vaccine. Bull. World Health Organ..

[48] Shantavasinkul P., Tantawichien T., Wilde H., Sawangvaree A., Kumchat A., Ruksaket N., Lohsoonthorn V., Khawplod P., Tantawichien T. (2010). Postexposure rabies prophylaxis completed in 1 week: Preliminary study. Clin. Infect. Dis..

[49] Warrell M.J., Riddell A., Yu L.M., Phipps J., Diggle L., Bourhy H., Deeks J.J., Fooks A.R., Audry L., Brookes S.M. (2008). A simplified 4-site economical intradermal post-exposure rabies vaccine regimen: A randomised controlled comparison with standard methods. PLoS Negl. Trop. Dis..

[50] Quiambao B.P., Ambas C., Diego S., Bosch Castells V., Korejwo J., Petit C., Houillon G. (2019). Intradermal post-exposure rabies vaccination with purified Vero cell rabies vaccine: Comparison of a one-week, 4-site regimen versus updated Thai Red Cross regimen in a randomized non-inferiority trial in the Philippines. Vaccine.

[51] Tantawichien T., Jaijaroensup W., Khawplod P., Sitprija V. (2001). Failure of multiple-site intradermal postexposure rabies vaccination in patients with human immunodeficiency virus with low CD4+ T lymphocyte counts. Clin. Infect. Dis..

[52] RAC17 (2002). Clinical Study Report. Combined immunogenicity of Chromatographically Purified Rabies Vaccine (CPRV, with and without Merthiolate) and equine rabies immune globulin (ERIG), in comparison with Purified Vero Rabies Vaccine (PVRV) and ERIG, administered by intradermal route, in subjects with a WHO Category I, II, or III Rabies exposure.

[53] Cantaert T., Borand L., Kergoat L., Leng C., Ung S., In S., Peng Y., Phoeun C., Hing C., Taing C.N. (2019). A 1-week intradermal dose-sparing regimen for rabies post-exposure prophylaxis (RESIST-2): An observational cohort study. Lancet Infect. Dis..

[54] Buchy P., Preiss S., Singh V., Mukherjee P. (2017). Heterogeneity of Rabies Vaccination Recommendations across Asia. Trop. Med. Infect. Dis..

[55] World Health Organization (2018). WHO Expert Consultation on Rabies, Third Report: WHO Technical Report Series, No. 1012. https://apps.who.int/iris/bitstream/handle/10665/272364/9789241210218-eng.pdf.

[56] Kessels J., Tarantola A., Salahuddin N., Blumberg L., Knopf L. (2019). Rabies post-exposure prophylaxis: A systematic review on abridged vaccination schedules and the effect of changing administration routes during a single course. Vaccine.

[57] Angsuwatcharakon P. (2018). Neutralizing Immunogenicity Antibody After 2 Intradermal Doses Pre-Exposure Prophylaxis of Purified Vero Cell Rabies Vaccine (PVRV) to 12–24 month-old Children, concomitantly with Chimeric Live-Attenuated JE Vaccine (IMOJEV).

[58] Langedijk A.C., De Pijper C.A., Spijker R., Holman R., Grobusch M.P., Stijnis C. (2018). Rabies Antibody Response After Booster Immunization: A Systematic Review and Meta-analysis. Clin. Infect. Dis..

[59] Kessels J.A., Recuenco S., Navarro-Vela A.M., Deray R., Vigilato M., Ertl H., Durrheim D., Rees H., Nel L.H., Abela-Ridder B. (2017). Pre-exposure rabies prophylaxis: A systematic review. Bull. World Health Organ..

[60] Hampson K., Abela-Ridder B., Bharti O., Knopf L., Lechenne M., Mindekem R., Tarantola A., Zinsstag J., Trotter C. (2019). Modelling to inform prophylaxis regimens to prevent human rabies. Vaccine.

[61] Moore S.M., Hanlon C.A. (2010). Rabies-specific antibodies: Measuring surrogates of protection against a fatal disease. PLoS Negl. Trop. Dis..

