# Supplementary material for: Purified Vero Cell Rabies Vaccine (PVRV, Verorab®): A Systematic Review of Intradermal Use Between 1985 and 2019
Source: Trop Med Infect Dis. 2020 Mar 7;5(1):40. doi: 10.3390/tropicalmed5010040 (PMC7157209; doi:10.3390/tropicalmed5010040)
Supplement: Supplementary file 1 [file tropicalmed-05-00040-s001.zip › Supplementary Table 1 (31 Jan 2020).docx]

Table 1: Summary of publications on pre-exposure rabies prophylaxis (PrEP) identified through a systematic review of the literature up to 2019

| **Lead author/year** | **Country** | **Detection methods** | **Study design** | **Population assessed** | **Vaccination days  (number of sites)** | **Volume per site; mL (potency)** | **Immunogenicity** |
| --- | --- | --- | --- | --- | --- | --- | --- |
| Sabchareon et al 1998 [1] | Thailand | RFFIT | RCT | Children aged 5–12 years with no history of previous rabies immunization | Group A: 0, 7, 28 (1-1-1) ID  Group B: 0, 7, 28 (1-1-1) IM  All received booster at 1 year as per randomized group | Group A, 0.1 mL  Group B, 0.5 mL  *PVRV 4.64 (primary series) and 7.26 IU/mL (booster) per 0.5 mL* | Group A (n=94)  *GMTs, IU/mL (95% CI) [range]/Seroconversion rate   - Day 56: 4.7 (4.0–5.5)[0.4–40.6]/98.9% - Day 180: 0.5 (0.4–0.6)[0.01–25.3]/50.6% - Day 365: 0.4 (0.3–0.5)[0.01–5.7]/52.5%   Post-booster   - Day 7 post-booster: 11.8 (9.8–14.3)[1.7–137.8]/100% - Day 180 post-booster: 2.3 (1.8–3.1)[0.01–28.5]/93.5% - Day 365 post-booster: 1.9(1.5–2.4) [0.3–37.3]/95.9% - Day 730 post-booster: 1.6 (1.2–2.0) [0.2–13.1]/88.3%   Group B (n=93)  *GMTs, IU/mL (95% CI) [range]/Seroconversion rate   - Day 56: 1.8 (9.2–12.6)[1.3–140.2]/100% - Day 180: 1.4 (1.1–1.7)[0.2–87.1]/91.0% - Day 365: 1.3 (0.2–49.1)[1.1–1.6]/90.7%   Post exposure booster   - Day 7 post-booster: 22.9 (19.2–27.4)[4.5–460.7]/100% - Day 180 post-booster: 6.0 (4.3–8.2)[0.01–73.9]/97.1% - Day 365 post-booster: 5.3 (4.0–6.9) [0.5–41.0]/100% - Day 730 post-booster: 3.5 (2.5–4.8) [0.01–49.9]/96.7% |
| Lang et al 1999 [2] | Vietnam | RFFIT | RCT | Health children 2–5 months. Infants with mothers who received rabies immunization in the previous 2 years were excluded | Group A: months of age 2, 3, 4 (1-1-1) ID  Group B: months of age 2, 3, 4 (1-0-1) IM  Concomitant with DTP-IPV injection | Group A, 0.1 mL  Group B, 0.5 mL  *Three PVRV batches used (randomized across the two groups): 3.5 IU/0.5 mL; 6.4 IU/0.5 mL; 12.0 IU/).5mL* | Group A (n=116)  GMTs, IU/mL (95% CI)/Seroconversion rate   - Day 0: 0.032 (0.027–0.038)/0% - Week 15: 12.0 (10.5–13.6)/100%   Group B (n=117)  GMTs, IU/mL (95% CI)/Seroconversion rate   - Day 0: 0.033 (0.028–0.039)/0% - Week 15: 30.6 (27.9–33.7)/100% |
| Tantawichien, et al 1999 [3] | Thailand | RFFIT | NA | Veterinary students who had all received a completed course PrEP rabies vaccination with PVRV (0, 7, 28 [1-1-1] ID 1 year earlier). None had received booster | 0, 7, 28 [1-1-1] ID 1 year earlier | 0.1 mL  *PVRV 11.8 IU/0.5 mL potency* | 38% (16/42) of all subjects had RVNA titers of ≥0.5 IU/mL 1 year after PrEP completion; GMT range 0.04–5.73 (GMTs reported as 0.4 and 0.43 in this population split to receive subsequent ID or IM PEP, respectively) |
| Khawplod et al. 2002 [4] | Thailand | RFFIT | Comparative study | Volunteers with no previous history of rabies vaccination (age not stated) | Group A (HDCV): 0, 7, 28 (1-1-1) IM with booster 0,3 (1-1) IM  Group B (HDCV): 0, 7, 28 (1-1-1) IM with booster 0,3 (4-0) ID  Group C (PCEC): 0, 7, 28 (1-1-1) IM with booster 0,3 (1-1) IM  Group D (PCEC): 0, 7, 28 (1-1-1) IM with booster 0,3 (4-0) ID  Group E (PCEC): 0, 7, 28 (1-1-1) ID with booster 0,3 (1-1) IM  Group F (PCEC): 0, 7, 28 (1-1-1) ID with booster 0,3 (4-0) ID  Group G (PVRV): 0, 7, 28 (1-1-1) IM with booster 0,3 (1-1) IM  Group H (PVRV): 0, 7, 28 (1-1-1) IM with booster 0,3 (4-0) ID  Group I (PVRV): 0, 7, 28 (1-1-1) ID with booster 0,3 (1-1) IM  Group J (PVRV): 0, 7, 28 (1-1-1) ID with booster 0,3 (4-0) ID  All booster at 1 year (Day 360) either ID or IM depending on group (shown) | Group A, 1.0mL  Group B, ID=0.1 mL  Group C, 1.0mL  Group D, ID=0.1 mL  Group E, ID=0.1 mL  Group F, ID=0.1 mL  Group G, not stated  Group H, ID=0.1 mL  Group I, ID=0.1 mL  Group J, ID=0.1 mL  IM volume not stated  *HDCV 4.7 IU/mL potency*  *PCEC 8.5 IU/mL potency*  *PVRV 8.7 IU/0.5 mL potency/* | Group A (n=40)  PrEP (IM)  GMTs, IU/mL [range] (Group A and B)/seroconversion rate   - Day 14: 4.90 [1.04–35.36]/100%   Post-exposure booster, IM (n=18)  GMTs, IU/mL [range]   - Day 0: 1.92 [0.4–5.9]/- - Day 7: 18.22 [5.7–32.0]/100% - Day 14: 36.25 [14.1–90.5]/100%   Group B (n=40)  PrEP (IM)  GMTs, IU/mL [range] (Group A and B)/seroconversion rate   - Day 14: 4.90 [1.04–35.36]/100%   Post-exposure booster, ID (n=19)  GMTs, IU/mL [range]/seroconversion rate   - Day 0: 1.36 [0.3–4.6]/- - Day 7: 44.24 [10.4–383.1]/100% - Day 14: 105.36 [38.1–247.6]/100%   Group C (n=37)  PrEP (IM)  GMTs, IU/mL [range] (Group C and D)/seroconversion rate   - Day 14: 5.29 [0.42–38.56]/-   Post-exposure booster, IM (n=18)  GMTs, IU/mL [range]/seroconversion rate   - Day 0: 0.70 [0.2–2.6]/- - Day 7: 14.18 [7.7–59.5]/100% - Day 14: 46.90 [11.5–545.3]/100%   Group D (n=37)  PrEP (IM)  GMTs, IU/mL (range) (Group C and D)/seroconversion rate   - Day 14: 5.29 (0.42–38.56)/-   Post-exposure booster, ID (n=19)  GMTs, IU/mL [range]/seroconversion rate   - Day 0: 0.97 [0.1–7.3]/- - Day 7: 34.50 [5.4–173.3]/100% - Day 14: 101 [32.4–351.3]/100%   Group E (n=39)  PrEP (ID)  GMTs, IU/mL [range] (Group E and F)/seroconversion rate   - Day 14: 2.37 [0.10–38.56]/-   Post-exposure booster, IM (n=19)  GMTs, IU/mL [range]/seroconversion rate   - Day 0: 0.31 [0.04–5.7]/- - Day 7: 14.27 [2.0–175.6]/100% - Day 14: 78.13 [4.8–766.1]/100%   Group F (n=39)  PrEP (ID)  GMTs, IU/mL [range] (Group E and F)/seroconversion rate   - Day 14: 2.37 [0.10–38.56]/-   Post-exposure booster, ID (n=19)  GMTs, IU/mL [range]/seroconversion rate   - Day 0: 0.34 [0.1–1.4]/- - Day 7: 15.15 [5.7–74.3]/100% - Day 14: 63.91 [10.7–244.1]/100%   Group G (n=44)  PrEP (IM)  GMTs, IU/mL [range] (Group G and H)/seroconversion rate   - Day 14: 7.08 [1.41–56.20]/100%   Post-exposure booster, IM (n=24)  GMTs, IU/mL [range)/seroconversion rate   - Day 0: 1.00 [0.1–8.5]/- - Day 7: 11.29 [2.0–79.5]/100% - Day 14: 30.47 [2.5–1110.8]/100%   Group H (n=44)  PrEP (IM)  GMTs, IU/mL [range] (Group G and H)/seroconversion rate   - Day 14: 7.08 [1.41–56.20]/100%   Post-exposure booster, ID (n=19)  GMTs, IU/mL [range]/seroconversion rate   - Day 0: 1.11 [0.4–5.3]/- - Day 7: 44.21 [12.5–135.4]/100% - Day 14: 143.4 [10.7–244.1]/100%   Group I (n=42)  PrEP (ID)  GMTs, IU/mL [range] (Group I and J)/seroconversion rate   - Day 14: 3.40 [0.16–39.74]/-   Post-exposure booster, IM (n=19)  GMTs, IU/mL [range]/seroconversion rate   - Day 0: 0.46 [0.2–1.1]/- - Day 7: 11.7 [2.9–248.4]/100% - Day 14: 55.99 [16.2–993.5]/100%   Group J (n=42)  PrEP (ID)  GMTs, IU/mL [range] (Group I and J)/seroconversion rate   - Day 14: 3.40 [0.16–39.74]/-   Post-exposure booster, ID (n=20)  GMTs, IU/mL [range]/seroconversion rate   - Day 0: 0.26 [0.06–3.5]/- - Day 7: 25.90 [4.6–86.7]/100% - Day 14: 79.49 [25.8–227.8]/100% |
| Khawplod et al 2007 [5] | Thailand | RFFIT | RCT | Volunteers aged 8 to 40 years with no history of previous rabies immunization | Group A: 0, 7, 28 (2-2-2) ID; booster 360, 363 (1-1) ID  Group B: 0, 3, 7 (2-2-2) ID; booster 360, 363 (1-1) ID  Group C: 0, 3, 7 (1-1-1) IM; booster 360, 363 (1-1) ID  Group D: 0, 3, 7 (2-0-0) ID; booster 360, 363 (1-1) ID  ID booster on days 360 and 363 for groups A–D  PEP regimens (Group E-F) summarized other summary table | Dose: Primary/booster  Group A: 0.1 mL ID/0.1 mL ID  Group B: 0.1 mL ID/0.1 mL ID  Group C: 1 mL IM/0.1 mL ID  Group D: 0.1 mL ID/0.1 mL ID  *PVRV 7.5 IU/0.5mL potency*  *Purified chick embryo vaccine 7.0 IU/0.5mL potency* | Group A (n=16)  GMTs, IU/mL [range]/Seroconversion rate   - Day 0: <0.03 [–]/–% - Day 360: 0.96 [0.32–2.95]/81%   Post-exposure booster   - Day 7: 29.14 [6.73–227.76]/100% - Day 14: 49.39 [13.05–308.44]/100%   Group B (n=16)  GMTs, IU/mL [range]/Seroconversion rate   - Day 0: <0.03 [–]/–% - Day 360: 1.12 [0.30–4.76)/94%   Post-exposure booster   - Day 7: 22.99 [5.91–295.37]/100% - Day 14: 105.08 [26.11–496.74]/100%   Group C (n=20)  GMTs, IU/mL [range)/Seroconversion rate   - Day 0: <0.03 [–)/–% - Day 360: 0.97 [0.27–4.76]/80%   Post-exposure booster   - Day 7: 35.16 [11.97–191.52]/100% - Day 14: 125.0 [29.73–800.0]/100%   Group D (n=14)  GMTs, IU/mL [range]/Seroconversion rate   - Day 0: <0.03 [–]/–% - Day 360: 0.41 [0.06–3.67]/38%   Post-exposure booster   - Day 7: 9.15 [3.36–22.63]/100% - Day 14: 51.96 [13.63–141.42]/100% |
| Vien et al 2008 [6] | Vietnam | RFFIT | RCT | Healthy children aged 16—20 months who had completed their primary series 1 year earlier (primary regimen not stated) | Group A: 1-site ID  Group B: 1-sites IM  All also received concomitant DTP and OPV in accordance with the Vietnamese pediatric vaccination schedule (See Lang et al 1999). Booster at Year 5 also. | Group A, 0.1 mL  Group B, 0.5 mL  *PVRV potency not stated* | Group A (n=115)  GMTs, IU/mL/seroconverted   - Day 0; 1.9 /95% - Day 14; 23/100% - Y1; 2.7/98% - Y2; 1.7/96% - Y3; 1.0/83% - Y4; 0.8/68% - Y5; 0.6/54% - Y5+14 days; 9.5/100%   Group B (n=113)  GMTs, IU/mL/seroconverted   - Day 0; 2.5 /97% - Day 14; 40/100% - Y1; 5.0/100% - Y2; 3.0/97% - Y3; 2.0/94% - Y4; 1.2/86% - Y5; 1.0/80% - Y5+14 days; 10.1/100%   Data estimated from graph |
| Cunha et al 2010 [7] | Brazil | RFFIT | RCT | Professionals aged ≥18 years at risk of exposure to the rabies virus, 18 years (without prior rabies immunization) | Group A: regimen not specified (Assumed 0, 7, 28 [1-1-1], ID  Group B: regimen not specified (Assumed 0, 7, 28 [1-1-1], IM | Group A, 0.1 mL  Group B, 0.5 mL  *PVRV ≥2.5 IU/mL per dose potency* | Group A (n=65)  GMTs, IU/mL/seroconversion   - Day 0; 0.1854/0% - Day 38; 1.9033/96.9% - Day 118; 0.7551/48.3% - Day 208; 0.5508/20.7%   Group B (n=62)  GMTs, IU/mL/seroconversion   - Day 0; 0.1871/0% - Day 38; 2.8573/100% - Day 118; 1.2040/89.5% - Day 208; 0.8929/63.5% |
| Wongsaroj et al 2013 [8] | Thailand | RFFIT | RCT | Seronegative healthy subjects aged between 18 and 24 years | Group A: 0,7, 21 (2-0-2) ID  Group B: 0, 7, 21 (1-1-1) IM  All received 0.1mL PVRV ID booster on D365 and D368 | Group A, 0.1 mL  Group B, 0.5 mL  *PVRV 4.8 IU/0.5mL potency* | Group A (n=39)  GMTs, IU/mL [range]   - Day 35; 4.51 [1.69–13.0]/100% seroconverted - Day 365; 0.35 [0.11–1.76] - Day 379 (14 days post-booster); 14.38 [2.99–308.44]   Group B (n=16)  GMTs, IU/mL [range]   - Day 35; 6.74 [2.30–14.23] - Day 365; 0.76 [0.18–2.83] - GMT Day 379 (14 days post-booster); 14.06 [3.12–62.09]   All participants in both groups had seroconverted on Day 35  All participants in both groups seroconverted Day 379 (14 days after booster) |
| Jonker et al 2017 [9] | The Netherlands | FAVN | RCT | Healthy Volunteers aged 18 to 31 years without previous rabies vaccination | Group A: 1-site ID  Group B: 2-sites ID  Group C: 3 sites ID  Group D: 1 site IM  All received booster after 1 year; 2 standard doses (0.5mL) in the ipsilateral deltoid muscle on day 0 and day 3 | Group A: 0.1 mL  Group B: 0.1 mL  Group C: 0.1 mL  Group D: 0.5 mL  *PVRV 3.2 IU/0.5mL potency* | Group A (n=10)  GMTs, IU/mL (95% CI) [range]/Seroconversion rate   - Day 7: 0 (–)[–]/–% - Day 28: 2.0 (1.1–3.8)[0.4–7.4]/90% - Pre-booster: 0 (0.0–2.0)[0.1–1.3]/20% - Day 3 post-booster: 0 (0.0–1.5)[0.1–1.3]/–% - Day 7 post-booster: 22.6 (10.8–47.0) [3.0–239.2]/100%   Group B (n=5)  GMTs, IU/mL (95% CI) [range]/Seroconversion rate   - Day 7: 0 (–)[–]/–% - Day 28: 6.7 (2.9–15.4)[2.5–29]/100% - Pre-booster: 0.2 (0.1–0.4)[0.1–0.6]/20% - Day 3 post-booster: 0.3 (0.1–0.6)[0.1–0.7]/–% - Day 7 post-booster: 13.0(7.7–22.0) [5.1–26.6]/100%   Group C (n=5)  GMTs, IU/mL (95% CI) [range]/Seroconversion rate   - Day 7: 0 (–)[–]/–% - 1 month: 4.2 (1.4–13.0)[0.8–16.8]/100% - Pre-booster: 0.5 (0.3–0.8)[0.2–1.0]/40% - Day 3 post-booster: 0.5 (0.3–0.9)[0.2–1.3]/–% - Day 7 post-booster: 20.1(12.9–31.5) [11.7–34.8]/100%   Group D (n=10)  GMTs, IU/mL (95% CI) [range]/Seroconversion rate   - Day 7: 0 (–)[–]/–% - 1 month: 2.2 (1.3–3.9)[0.5–11.6]/90% - Pre-booster: 0.4(0.2–0.6)[0.1–1.3]/30% - Day 3 post-booster: 0.3 (0.2–0.6)[0.1–1.3]/–% - Day 7 post-booster: 63.9 (45.1–90.6) [26.6–239.2]/100% |
| De Pijper et al 2018 [10] | The Netherlands | RFFIT | Observational cohort study | Dutch military personnel (without prior rabies immunization) aged 18–48 years | *0, 7 (1-1) ID* | *0.1 mL*  *PVRV potency not stated* | Eligible data (n=430); data presented for those who seroconverted (n=340) at Day 21 and (n=90) at Day 28, and both days combined.  GMTs, IU/mL (95% CI) [range]/Seroconversion rate   - Day 21; 6.25 (5.80–6.74)[0.79–49.99]/99.1% - Day 28; 15.65 (13.84–17.70) [3.19–112.51]/100% - Day 21/28; 7.59 (7.04–8.17)[0.79–112.52/99.3% |
| Angsuwatcharakon et al 2019 [11] | Thailand | RFFIT | RCT | Healthy children aged 12–16 months with no history of previous rabies immunization or titer ≥0.5IU/mL | Group A: 0, 7, 28 (2-0-2) ID  Group B: 0, 7, 28 (1-1-1) IM  concomitantly with Chimeric Live-Attenuated JE Vaccine (IMOJEV) on D0 and D365 | Group A: 0.1 mL  Group B: 0.5 mL  *PVRV 4.0 IU/0.5mL potency* | Group A (n=32)  GMTs, IU/mL (95% CI) [range]/Seroconversion rate   - Day 0: not detectable/0% - Day 42: 14.35 (13.84–21.62) [2.10–43.62]/100% - Day 365: 1.50 (1.42–2.66) [0.15–6.16]/92.3%   Group B (n=17)  GMTs, IU/mL (95% CI) [range]/Seroconversion rate   - Day 0: not detectable/0% - Day 42: 14.83 (11.81–24.22) [4.78–41.77]/100% - Day 365: 2.00 (0.47–4.91) [0.47–4.91]/92.3% |

Data shown for days since first dose unless otherwise stated

Data shown for PVRV unless otherwise stated

Seroconversion ≥0.5 IU/ml (protective level) unless specified

*Data shown for Days after the primary immunization.

FAVN, Fluorescent Antibody Virus Neutralization; ID, intradermal; IM, intramuscular; PVRV, purified Vero cell rabies vaccine, RCT, randomized controlled study; RFFIT, rapid fluorescent focus inhibition test

**References**

1. Sabchareon, A.; Chantavanich, P.; Pasuralertsakul, S.; Pojjaroen-Anant, C.; Prarinyanupharb, V.; Attanath, P.; Singhasivanon, V.; Buppodom, W.; Lang, J. Persistence of antibodies in children after intradermal or intramuscular administration of preexposure primary and booster immunizations with purified Vero cell rabies vaccine. *Pediatr Infect Dis J* **1998**, *17*, 1001-1007, doi:10.1097/00006454-199811000-00007.

2. Lang, J.; Hoa, D.Q.; Gioi, N.V.; Vien, N.C.; Nguyen, C.V.; Rouyrre, N.; Forrat, R. Immunogenicity and safety of low-dose intradermal rabies vaccination given during an Expanded Programme on immunization session in Viet Nam: results of a comparative randomized trial. *Trans R Soc Trop Med Hyg* **1999**, *93*, 208-213, doi:10.1016/s0035-9203(99)90309-7.

3. Tantawichien, T.; Benjavongkulchai, M.; Limsuwan, K.; Khawplod, P.; Kaewchompoo, W.; Chomchey, P.; Sitprija, V. Antibody response after a four-site intradermal booster vaccination with cell-culture rabies vaccine. *Clin Infect Dis* **1999**, *28*, 1100-1103, doi:10.1086/514737.

4. Khawplod, P.; Benjavongkulchai, M.; Limusanno, S.; Chareonwai, S.; Kaewchompoo, W.; Tantawichien, T.; Wilde, H. Four-site intradermal postexposure boosters in previously rabies vaccinated subjects. *J Travel Med* **2002**, *9*, 153-155, doi:10.2310/7060.2002.23189.

5. Khawplod, P.; Wilde, H.; Benjavongkulchai, M.; Sriaroon, C.; Chomchey, P. Immunogenicity study of abbreviated rabies preexposure vaccination schedules. *J Travel Med* **2007**, *14*, 173-176, doi:10.1111/j.1708-8305.2007.00120.x.

6. Vien, N.C.; Feroldi, E.; Lang, J. Long-term anti-rabies antibody persistence following intramuscular or low-dose intradermal vaccination of young Vietnamese children. *Trans R Soc Trop Med Hyg* **2008**, *102*, 294-296, doi:10.1016/j.trstmh.2007.11.010.

7. Cunha, R.S.; Silva Ade, C.; Batista, A.M.; Chaves, L.B.; Barata, R.B. Equivalence between pre-exposure schemes for human rabies and evaluation of the need for serological monitoring. *Rev Saude Publica* **2010**, *44*, 548-554, doi:10.1590/s0034-89102010005000005.

8. Wongsaroj, P.; Udomchaisakul, P.; Tepsumethanon, S.; Khawplod, P.; Tantawichien, T. Rabies neutralizing antibody after 2 intradermal doses on days 0 and 21 for pre-exposure prophylaxis. *Vaccine* **2013**, *31*, 1748-1751, doi:10.1016/j.vaccine.2013.01.035.

9. Jonker, E.F.F.; Visser, L.G. Single visit rabies pre-exposure priming induces a robust anamnestic antibody response after simulated post-exposure vaccination: results of a dose-finding study. *J Travel Med* **2017**, *24*, doi:10.1093/jtm/tax033.

10. De Pijper, C.A.; Boersma, J.; Terryn, S.; Van Gucht, S.; Goorhuis, A.; Grobusch, M.P.; Stijnis, C. Rabies antibody response after two intradermal pre-exposure prophylaxis immunizations: An observational cohort study. *Travel Med Infect Dis* **2018**, *22*, 36-39, doi:10.1016/j.tmaid.2018.03.006.

11. Angsuwatcharakon, P.; Ratananpinit, N.; Yoksan, S.; Saengseesom, W.; Sriaksorn, R.; Raksahket, N.; Tantawichien, T. Immunogenicity and safety of a double-dose, two-visit, pre-exposure rabies prophylaxis regimen versus a conventional regimen with Vero cell rabies and concomitant chimeric live-attenuated Japanese encephalitis vaccine administration. *Vaccine* **2019**, *(Submitted 2019)*.
