# Supplementary material for: Purified Vero Cell Rabies Vaccine (PVRV, Verorab®): A Systematic Review of Intradermal Use Between 1985 and 2019
Source: Trop Med Infect Dis. 2020 Mar 7;5(1):40. doi: 10.3390/tropicalmed5010040 (PMC7157209; doi:10.3390/tropicalmed5010040)
Supplement: Supplementary file 1 [file tropicalmed-05-00040-s001.zip › Supplementary Table 2 (31 Jan 2020).docx]

Table 2: Summary of publications on post-exposure rabies prophylaxis (PEP) identified through a systematic review of the literature up to 2019

| **Lead author/year** | | **Country** | | **Detection methods** | | | **Study design** | | | | **Population assessed** | | | **Vaccination days  (number of sites)** | **Volume per site; mL (potency)** | | | **Immunogenicity** | |
| --- | --- | --- | --- | --- | --- | --- | --- | --- | --- | --- | --- | --- | --- | --- | --- | --- | --- | --- | --- |
| Phanuphak et al 1987 [1] | | Thailand | | RFFIT | | | RCT | | | | Health subjects (aged 14–58 years) with low risk of exposure to rabies (bitten by runaway dog or licked by a rabid dog (not previously immunized) | | | Group A: 0, 3, 7, 14, 28 (4-4-4-0-1) ID  Group B: 0, 3, 7, 14, 28 (2-2-2-0-1) ID  Group C: 0, 3, 7, 14, 28 (1-1-1-0-1) ID  Group D: 0, 3, 7, 14, 28 (1-1-1-1-1) IM | Group A, 0.1 mL ID  Group B, 0.1 mL ID  Group C, 0.1 mL ID  Group D, 0.5 mL IM  *PVRV 5.1 IU/0.5mL potency* | | | Group A (n=14)  GMTs, IU/mL [range]/seroconversion rate   - Day 0: 0 - Day 3: 0 - Day 7: 0.02 [0–4.43]/14.2% - Day 14: 12.66 [2.30–235.22]/100% - Day 28: 7.76 [3.20–24.92]/100% - Day 35: 10.73 [3.30–40.05]/100%   Group B (n=15)  GMTs, IU/mL [range]/Seroconversion rate   - Day 0: 0 - Day 3: 0 - Day 7: 0.01 [0–0.45]/0.0% - Day 14: 5.79 [0.69–22.40]/100% - Day 28: 5.07 [1.12–12.10]/100% - Day 35: 4.69 [0.85–25.86]/100%   Group C (n=14)  GMTs, IU/mL [range]/Seroconversion rate   - Day 0: 0 - Day 3: 0 - Day 7: 0 - Day 14: 2.76 [0.19–10.36]/85.7% - Day 28: 3.38 [0.75–20.80]/100% - Day 35: 4.96 [1.50–10.96]/100%   Group D (n=15)  GMTs, IU/mL [range]/Seroconversion rate   - Day 0: 0 - Day 3: 0 - Day 7: 0 - Day 14: 5.95 [0.6–22.4]/100% - Day 28: 5.95 [1.6–19.0]/100% - Day 35: 8.99 [4.2–20.9]/100% | |
| Chutivongse et al 1990 [2] | | Thailand | | RFFIT | | | Observational | | | | Patients (aged 2 months to 60 years) severely bitten by rabid animal with no history of previous rabies vaccination | | | 0, 3, 7, 14, 30, 90 (2-2-2-0-1-1) ID  All received ERIG (40 IU/kg ERIG for 96 subjects or 20 IU HRIG for 4 subjects) | 0.1 mL ID  *PVRV 3.17 IU/ 0.5mL dose potency* | | | Antibody was measured in 10 randomly selected adults  GMTs, IU/mL [range]/Seroconversion rate   - Day 14: 3.475 [1.05–6.94]/100% - Day 90: 0.938 [0.55–3.11]/100% - Day 360: 0.738 [0.55–3.0]/100% | |
| Phanuphak et al 1990 [3] | | Thailand | | RFFIT | | | RCT | | | | Health children and adults (aged 14-55 years) who petted or been licked by a rabid animal on unbroken skin (but did not need rabies vaccination–prior vaccination history not stated) | | | Group A: 0, 3, 7 (2-2-2) ID  Group B: 0, 3, 7 (2-2-2) ID and SC (one dose each)  Group A: 0, 3, 7 (2-2-2) SC | Group A, 0.1 mL ID  Group B, 0.1 mL (ID and SC each 0.1mL)  Group C, 0.1 mL SC  *PVRV 10.35 IU/0.5mL potency* | | | Group A (n=15)  GMTs, IU/mL [range]/Seroconversion rate   - Day 14: 15.5 [3.9–41.9]/100%   Group B (n=14)  GMTs, IU/mL [range]/Seroconversion rate   - Day 14: 26.4 [5.3–50.9]/100%   Group C (n=15)  GMTs, IU/mL [range]/Seroconversion rate   - Day 14: 18.5[3.3–41.2]/100% | |
| Kositprapa et al 1997 [4] | | Thailand | | RFFIT | | | Comparative study | | | | Veterinary students who had all received a completed course PrEP rabies vaccination with PVRV (0, 7, 28 [1-1-1] ID 12–16 months earlier). | | | Group A: 0, 3 (1-1) ID  Group B: 0, 3 (1-1) IM  Route of vaccine administration the same as for PrEP | Group A, 0.1 mL  Group B, 0.5 mL  *PVRV 6.0 IU/mL potency* | | | Group A (n=35)  GMTs, IU/mL [range]/Seroconversion rate   - Day 0: 0.20 [0.03–2.20]/20% - Day 7: 4.30 [0.42–31.05]/97% - Day 14: 10.78 [2.04–161.05]/100%   Group B (n=31)  GMTs, IU/mL [range]/Seroconversion rate   - Day 0: 0.57 [0.04–11.54]/39% - Day 7: 10.24 [2.65–59.85]/100% - Day 14: 26.76 [5.03–218.1]/100% | |
| Tantawichien, et al 1999 [5] | | Thailand | | RFFIT | | | RCT | | | | Veterinary students who had all received a completed course PrEP rabies vaccination with PVRV (0, 7, 28 [1-1-1] ID 1 year earlier). None had received booster) | | | Group A: 0, 3 (4-0) ID  Group B: 0, 3 (1-1) IM | Group A, 0.1 mL  Group B, 0.5 mL  *PVRV 8.7 IU/0.5 mL potency* | | | 38% of all subjects had RVNA titers of ≥0.5 IU/mL 1 year after PrEP  All subjects  Group A (n=20)  GMTs, IU/mL [range]/Seroconversion rate   - Day 0: 0.40 [0.04–3.67]/40% - Day 3: 0.41 [0.05–2.59]/50% - Day 5: 1.42 [0.24–4.36]/95% - Day 14: 76.38 [17.56–417.7]/100% - Day 150: 19.20 [4.01–336.3]/100% - Day 360: 8.62 [1.62–200]/100%   Group B (n=22)  GMTs, IU/mL [range)/Seroconversion rate   - Day 0: 0.43 [0.15–5.73]/37% - Day 3: 0.41 [0.12–5.03]/45% - Day 5: 0.82 [0.21–8.11]/77% - Day 14: 12.18 [2.97–100]/100% - Day 150: 3.39 [1.14–16.10]/100% - Day 360: 1.86 [0.19–7.34]/95%   Subjects with RVNA <0.5 IU/mL before booster  Group A (n=12)  GMTs, IU/mL [range]/Seroconversion rate   - Day 0: 0.19 [0.04–0.48]/0% - Day 3: 0.22 [0.05–1.24]/ –% - Day 5: 1.09 [0.24–3.22]/–% - Day 14: 72.96 [17.56–417.7]/100% - Day 150: 15.78 [4.2–336.3]/100% - Day 360: 7.3 [1.62–33.63]/100%   Group B (n=14)  GMTs, IU/mL (range]/Seroconversion rate   - Day 0: 0.27 [0.15–0.42]/0% - Day 3: 0.28 [0.17–0.59]/–% - Day 5: 0.60 [0.21–1.24]/–% - Day 14: 8.61 [2.97–43.91]/100% - Day 150: 3.16 [1.14–16.10]/100% - Day 360: 1.55 [0.19–6.21]/–% | |
| Briggs et al 2000 [6] | | Thailand | | RFFIT | | | RCT | | | | Patients (aged 2–78 years) with category II and III exposure without a history of rabies vaccination | | | Group A (PVRV): 0, 3, 7, 14, 30, 90 (2-2-2-0-1-1) ID  Group B (PCECV): 0, 3, 7, 14, 30, 90 (2-2-2-0-1-1) ID  Group C (PCECV): 0, 3, 7, 14, 30, 90 (1-1-1-1-1-1) IM  Cat III received HRIG 20 IU/kg) | Group A, 0.1 mL ID  Group B, 0.1 mL ID  Group C, 0. 5 mL IM  *PVRV 11.6 IU/0.5 mL dose potency*  *PCECV 9.16 IU/1.0 mL dose potency* | | | Group A (n=59)  GMTs, IU/mL [range]/Seroconversion rate   - Day 7: 0.32 [0.1–2.2]/- - Day 14: 28.9 [1.6–350.0]/100% - Day 30: 10.9 [0.6–157.0]/100% - Day 90: 2.7 [0.5–47.0]/100%   Group B (n=58)  GMTs, IU/mL [range]/Seroconversion rate   - Day 7: 0.34 [0.05–19.1]/- - Day 14: 28.5 [1.1–1318.0]/100% - Day 30: 10.9 [1.5–171.0]/100% - Day 90: 3.0 [0.4–59.1]/-   Group C (n=37)  GMTs, IU/mL [range)/Seroconversion rate   - Day 7: 0.29 [<0.05–19.1]/- - Day 14: 12.3 [0.4–301.0]/95% - Day 30: 18.5 [0.5–217.0]/100% - Day 90: 4.7 [0.5–60.9]/100%   Data presented Cat II/III combined (i.e.. with and without HRIG combined data) | |
| Tantawichien et al 2001a [7] | | Thailand | | RFFIT | | | Comparative study | | | | Adults aged (17–42 years) who had received either PrEP- or PEP, without rabies immunoglobulin 1–10 years earlier | | | Group A: 0 (4) ID PVRV  Group B: 0 (4) ID PCECRV | Group A, 0.1 mL  Group B, 0.1 mL  *PVRV 10.8 IU/mL potency*  *PCECV 7 IU/mL potency* | | | Group A (n=10)  GMTs, IU/mL [range]/Seroconversion rate   - Day 0: not specified [0.14–3.38]/70% - Day 360: not specified [2.41–35.60]/100%   Group B (n=10)  GMTs, IU/mL [range]/Seroconversion rate   - Day 0: not specified [0.27–34.90]/80% - Day 360: not specified [2.03–243.37]/100% | |
| Tantawichien et al 2001b [8] | | Thailand | | | RFFIT | | | Observational | | HIV-infected patients (aged 7–43 years) without signs of active opportunistic infections who presented with possible or proven rabies exposure | | 0, 3, 7, 14, 30, 90 (4-4-4-0-2-2)  9/10 patients WHO category III exposure received HRIG administered as 20 IU/kg body weight | | | 0.1 mL  *PVRV 7.4 IU/mL potency* | | | n=10  Mean Titer, IU/mL [range]/Seroconversion rate   - Day 0: [<0.04–0.1]]/0% - Day 7: 0.46 [0.05–2.71]1/10% - Day 14: 3.97 [<0.04–10.37]/70% - Day 30: 2.34 [<0.04–8.35]/78% - Day 90: 1.96 [<0.04–5.66]/71% | |
| Khawplod et al 2002 [9] | | Thailand | | | RFFIT | | | RCT | | Healthy volunteers with no history of rabies vaccination (young, healthy veterinary students, nurses, or scientists) | | Group A: 0, 3, 7 (8-0-4) ID  Group B: 0, 3, 7 (4-4-4) ID  Group C: 0, 3, 7 (2-2-2) ID  Group D: 0, 3, 7,14 (1-1-1-1) IM  All received booster at 1 year (route not specified) | | | Group A, 0.1 mL  Group B, 0.1 mL  Group C, 0.1 mL  Group D, 0.5 mL^#^  *PVRV 6.9 IU/0.5mL potency* | | | Group A (n=22)  GMTs, IU/mL [range]/Seroconversion rate   - Day 0: <0.06 [–]/0% - Day 5: <0.06 [–]/0% - Day 7: 0.13 [<0.06–0.50]/4.5% - Day 14: 13.09 [4.97–30.64]100%   Group B (n=21)  GMTs, IU/mL [range]/Seroconversion rate   - Day 0: <0.06 [–]/0% - Day 5: <0.06 [<0.06–0.07]/0% - Day 7: 0.14 [<0.06–0.57]/9.5% - Day 14: 12.24 [2.18–24.68]/100%   Group C (n=21)  GMTs, IU/mL [range]/Seroconversion rate   - Day 0: <0.06 [–]/0% - Day 5: <0.06 [–]/0% - Day 7: 0.08 [<0.06–0.3]/0% - Day 14: 5.00 [1.76–12.88]/100%   Group D (n=21)  GMTs, IU/mL [range]/Seroconversion rate   - Day 0: <0.06 [–]/0% - Day 5: <0.06 [–]/0% - Day 7: 0.06 [<0.06–0.10]/0% - Day 14: 3.81 [0.71–12.88]/100% | |
| Madhusudana et al 2004 [10] | | India | | MNT | | | Comparative | | | | Health adults (aged 25–52 years) working in infectious diseases hospital and neurological intensive care units | | | Group A (PVRV): 0, 3, 7, 14, 28, 90 (2-2-2-0-1-1) ID  Group B (PCECV): 0, 3, 7, 14, 28, 90 (2-2-2-0-1-1) ID | Group A, 0.1 mL ID  Group B, 0.1 mL ID  *PVRV >2.5 IU/0.5 mL potency used*  *PCECV >2.5 IU/mL potency used* | | | Group A (n=38)  Mean titers, IU/mL (±SD)/ Seroconversion rate   - Day 10: 5.9±3.2/100% - Day 28: 12.1±4.5/100% - Day 90: 14.3±2.7/100% - Day 180: 5.9±2.5/100%   Group B (n=42)  Mean titers, IU/mL (±SD)/ Seroconversion rate   - Day 10: 5.8±3.1/100% - Day 28: 10.9±4.1/100% - Day 90: 13.1±3.9/100% - Day 180: 5.7±3.1/100%   Titers above 0.5 IU/ml of serum on all days tested. | |
| Ambrozaitis et al 2006 [11] | | Lithuania | | RFFIT | | | RCT | | | | Healthy subjects (aged 18– 60 years), with no history of rabies vaccination or history of animal bites | | | Group A (PVRV): 0, 3, 7, 14, 30, 90 (4-0-2-0-1-1) ID  Group B (PCECV): 0, 3, 7, 14, 30, 90 (4-0-2-0-1-1) ID | Group A, 0.1 mL ID  Group B, 0.1 mL ID  *PVRV 8.93 IU/ 0.5mL ID dose potency*  *PCECV 5.53 IU/1.0 mL ID dose potency* | | | Group A (n=87)  GMTs, IU/mL [range]/Seroconversion rate   - Day 7: 0.08 [<0.025–0.80]/6% - Day 14: 26.1 [3.83–95.9]/100% - Day 90: 2.75 [0.46–17.5]/99% - Day 104: 12.9 [0.70–181]/100%   Group B (n=86)  GMTs, IU/mL [range)/Seroconversion rate   - Day 7: 0.15 [<0.025–0.80]/3% - Day 14: 20.5 [3.42–71.2]/100% - Day 90: 2.39 [0.46–16.5]/99% - Day 104: 16.1 [1.0–320]/100% | |
| Khawplod et al 2006 [12] | | Thailand | | RFFIT | | | RCT | | | | Patients with category III rabies exposures and had no prior history of rabies vaccination | | | Group A (PVRV): 0, 3, 7, 14, 28, 90 (2-2-2-0-1-1) ID  Group B (CPRV): 0, 3, 7, 14, 28, 90 (2-2-2-0-1-1) ID  Group C (CPRV): 0, 3, 7, 14, 28, 90 (2-2-2-0-2-0) ID  All received HRIG (20 IU/kg) | Group A, volume not stated ID  Group B, volume not stated ID  Group C, volume not stated ID  *PVRV 7.1 IU/0.5mL dose potency*  *CPRV 5.2 IU/0.5mL dose potency* | | | Group A (n=105)  GMTs, IU/mL [range)/Seroconversion rate   - Day 7: 0.35 [0.29–0.42]/21% - Day 14: 10.8 [8.93–13.0]/100% - Day 28: 5.53 [4.62–6.64]/100% - Day 90: 2.32 [1.98–2.72]/100% - Day 180: 2.12 [1.77–2.55]/98% - Day 360: 1.09 [0.90–1.33]/80%   Group B (n=104)  GMTs, IU/mL [range)/Seroconversion rate   - Day 7: 0.35 [0.28–0.42]/14% - Day 14: 10.5 [8.63–12.8]/100% - Day 28: 6.52 [5.40–7.87]/100% - Day 90: 2.86 [2.39–3.41]/99% - Day 180: 2.40 [2.03–2.84]/99% - Day 360: 1.30 [1.09–1.55]/87%   Group C (n=107)  GMTs, IU/mL (range)/Seroconversion rate   - Day 7: 0.34 [0.29–0.39]/16% - Day 14: 8.86 [7.34–10.7]/100% - Day 28: 5.58 [4.61–6.76]/99% - Day 90: 3.32 [2.80–3.93]/99% - Day 180: 1.22 [0.99–1.48]/86% - Day 360: 0.72 [0.58–0.90]/65% | |
| Madhusudana et al 2006 [13] | | India | | RFFIT | | | RCT | | | | Subjects aged 5–60 years with category III exposure to suspect or proven rabid animals, not more than 48 hours ago, without having received previous rabies vaccinations | | | Group A (PVRV): 0, 3, 7, 14, 30, 90 (2-2-2-0-1-1) ID  Group B (PCECV): 0, 3, 7, 14, 30, 90 (2-2-2-0-1-1) ID  All received ERIG | Group A, 0.1 mL ID  Group B, 0.1 mL ID  *PVRV potency not stated*  *PCECV potency not stated* | | | Group A (n=50)  GMTs, IU/mL (95% CI)/seroconversion rate   - Day 14: 4.6 (4.4–4.9)/100% - Day 30: 8.7 (8.1–9.4)/100% - Day 90: 6.8 (6.5–7.1)/100% - Day 180: 3.6 (3.4–4.0)/100%   Group B (n=55)  GMTs, IU/mL (95% CI)/seroconversion rate   - Day 14: 4.3 (4.1–4.6)/100% - Day 30: 9.0 (8.4–9.6)/100% - Day 90: 6.7 (6.4–6.9)/100% - Day 180: 3.7 (3.4–4.0)/100% | |
| Khawplod et al 2007 [14] | Thailand | | | RFFIT | | | RCT | | Volunteers aged 8 to 40 years with no history of previous rabies immunization | | PrEP regimens (Group A-D) summarized in accompanying summary table  Group E: 0, 3, 7, 28, 90 (2-2-2-1-1) ID  Group F: 0, 3, 7, 28, 90 (2-2-2-1-1) ID-purified chick embryo vaccine | | | Group E: 0.1 mL ID/0.1 mL ID  Group F: 0.1 mL ID/0.1 mL ID  *PVRV 7.5 IU/0.5mL potency*  *Purified chick embryo vaccine 7.0 IU/mL potency* | | Group E (n=10)  GMTs, IU/mL [range]/Seroconversion rate   - Day 0: <0.03 [–]/–% - Day 7: 0.06 [0.03–0.15]/0% - Day 14: 5.56 [1.15–53.82]100% - Day 28: 5.84 [1.77–20.73]/100%   Group F (n=20)  GMTs, IU/mL [range)/Seroconversion rate   - Day 0: <0.03 [–]/–% - Day 7: 0.06 [<0.03–0.31]/0% - Day 14: 8.54 [1.56–27.26]/100% - Day 28: 5.96 [0.96–18.22]/100% | | |  |
| Quiambao et al 2008 [15] | | Philippines | | RFFIT | | | RCT | | | | Healthy subjects aged 5–50 years with category I or category II exposures to rabies (with no previous history of rabies vaccination) | | | Group A: 0, 3, 7, 14, 28, 90 (8-0-4-0-1-1) ID  Group B: 0, 3, 7, 14, 28, 90 (4-0-2-0-1-1) ID  Group C: 0, 3, 7, 14, 28, 90 (1-1-1-1-1-0) IM  Group D: 0, 3, 7, 14, 28, 90 (2-2-2-0-1-1) ID (plus RIG [offer ERIG unless positive skin test, then HRIG)  Booster administered 1 year after PEP (one dose) | Group A, 0.1 mL ID  Group B, 0.1 mL ID  Group C, 0.5 mL IM  Group D, 0.1 mL ID  *PVRV ≥2.5 IU/0.5mL potency* | | | Group A (n=96)  Seroconversion rate (%)   - Day 0: 0 - Day 5: 0 - Day 7: 5.2% - Day 14: 100% - Day 28: 100% - Pre-booster: 93.4% - Post-booster D14: 100%   Group B (n=96)  Seroconversion rate (%)   - Day 0: 0 - Day 5: 0 - Day 7: 1.04% - Day 14: 100% - Day 28: 100% - Pre-booster D14: 84.7% - Post-booster: 100%   Group C (n=97)  Seroconversion rate (%)   - Day 0: 0 - Day 5: 0 - Day 7: 1.04% - Day 14: 100% - Day 28: 100% - Pre-booster: 81.3% - Post-booster: 100%)   Group D (n=99)  Seroconversion rate (%)   - Day 0: 0 - Day 5: 0 - Day 7: 0 - Day 14: 100% - Day 28: 100% - Pre-booster: 78.7% - Post-booster: 100%   GMTs presented in a figure only (difficult to extract). | |
| Warrell et al 2008 [16] | | UK | | RFFIT and FAVN | | | RCT | | | | Healthy adults (18–51 years) without prior rabies vaccination | | | Group A: 0, 3, 7, 14, 28, 90 (4-0-2-0-1-1) ID  Group B: 0, 3, 7, 14, 28, 90 (8-0-4-0-1-1) ID  Group C: 0, 3, 7, 14, 28, 90 (2-2-2-0-1-1) ID  Group D: 0, 3, 7, 14, 28, 90 (1-1-1-1-1-0) IM | Group A, 0.1 mL ID  Group B, for 8-site (0.05 mL) but otherwise 0.1 mL ID  Group C, 0.1 mL ID  Group D, 0. 5 mL IM  *PVRV 5.3 IU/0.5 mL in 165 subjects and 8.4 IU/dose in 64 subjects dose potency* | | | Group A (n=55)  GMTs, IU/mL (95% CI) [range]/seroconversion rate   - Day 7: 0.44 (0.31–0.61)[0.02–6.89]/- - Day 14: 335 (279–402)[43.7–811.5]/100% - Day 90: 7.18 (5.63–9.15)[0.9–29.5]/100% - Day 360: 2.52 (1.79–3.55)[0.35–58.34]/-   Group B (n=58)  GMTs, IU/mL (95% CI) [range]/seroconversion rate   - Day 7: 0.67 (0.53–0.86)[0.06–6.21]/- - Day 14: 308 (249–382)[24.3–1459.0/100% - Day 90: 9.75 (7.46–12.76)[0.9–153.1]/100% - Day 360: 3.21 (2.38–4.34)[0.36–31.71]/-   Group C (n=58)  GMTs, IU/mL (95% CI) [range]/seroconversion rate   - Day 7: 0.88 (0.69–1.11)[0.02–8.39]/- - Day 14: 364 (299–442)[60.9–3712]/100% - Day 90: 9.14 (6.86–12.20)[1.2–229]/100% - Day 360: 4.6 (3.31–6.42)[0.62–295]/100%   Group D (n=56)  GMTs, IU/mL (95% CI) [range]/seroconversion rate   - Day 7: 0.36 (0.26–0.50)[0.02–2.79]/- - Day 14: 228 (162–322)[5.5–1283]/100% - Day 90: 6.21 (4.86–7.95)[0.9–81.9]/100% - Day 360: 1.33 (1.05–1.69)[0.10–6.32]/- | |
| Sampath et al 2010 [17] | | India | | RFFIT | | | RCT | | | | Healthy subjects (aged 13–59 years) not previously exposed to rabies and not taken any rabies vaccine in the past | | | Group A: 0, 3, 7, 14, 28 (2-2-2-0-2) ID (PVRV)  Group B: 0, 3, 7 (4-4-4) ID (Indirab) | Group A, volume not stated ID  Group B, volume not stated ID  *PVRV >2.5 IU/ dose potency*  *Indirab 5.7 IU/dose potency* | | | Group A (n=63)  GMTs, IU/mL (95% CI)   - Day 14: 3.26 (3.14–3.76) - Day 28: 7.70 (7.43–8.36) - Day 90: 3.57 (3.43–4.06)   Group B (n=67)  GMTs, IU/mL (95% CI)   - Day 14: 3.36 (3.26–3.96) - Day 28: 7.83 (7.57–8.51) - Day 90: 3.62 (3.43–4.12) | |
| Shantavasinkul et al 2010 [18] | | Thailand | | RFFIT | | | Comparative | | | | Healthy adults and those with category III exposure (range 19–58 years) without prior history of rabies vaccination | | | Group A: 0, 3, 7, 14, 28, 90 (4-4-4-0-0-0) ID  Group B : 0, 3, 7, 14, 28, 90 (4-4-4-0-0-0) ID + ERIG (40 IU/kg)  Group C with category III exposure: 0, 3, 7, 14, 28, 90 (2-2-2-0-1-1) ID + ERIG (40 IU/kg) | Group A, 0.1 mL ID  Group B, 0.1 mL ID  Group C, 0.1 mL ID  *PVRV 4.8 IU/0.5 mL dose potency* | | | Group A (n=45)  GMTs, IU/mL (95% CI) [range]/seroconversion   - Day 0: <0.03/0% - Day 7: 0.10 (0.07–0.13)[<0.03–0.86]/11.1% - Day 14: 18.58 (14.21–24.29)[3.44–104.43]/100% - Day 28: 8.61 (6.90–10.74)[2.77–67.71]/100% - Day 90: 2.18 (1.76–2.69)[0.65–25.0]/100% - Day 180: 1.41 (1.09–1.84)[0.34–25.0]/93.2% - Day 360: 1.17 (0.91–1.5)[0.27–10.0]/88.4%   Group B (n=45)  GMTs, IU/mL (95% CI) [range]/seroconversion   - Day 0: <0.03/0% - Day 7: 0.08 (0.06–0.11)[<0.03–3.88]/4.4% - Day 14: 19.68 (15.45–25.06)[4.82–197.0]/100% - Day 28: 9.32 (7.31–11.87)[1.35–135.4]/100% - Day 90: 1.83 (1.47–2.28)[0.30–15.0]/95.3% - Day 180: 1.26 (1.0–1.59)[0.30–15.0]/90.5% - Day 360: 1.05 (0.85–1.31)[0.30–5.34]/85%   Group C (n=41)  GMTs, IU/mL (95% CI) [range]/seroconversion   - Day 0: <0.03/0% - Day 7: 0.06 (0.05–0.08)[<0.03–0.68]/2.4% - Day 14: 7.39 (5.62–9.72)[1.22–31.05]/100% - Day 28: 4.06 (3.09–5.35)[0.78–19.9]/100% - Day 90: 1.76 (1.40–2.22)[0.27–8.05]/92.7% - Day 180: 2.23 (1.69–2.95)[0.34–16.37]/97.6% - Day 360: 1.29 (0.97–1.72)[0.23–8.84]/87.8% | |
| Tanisaro et al 2010 [19] | | Thailand | | RFFIT | | | Observational | | | | Hemodialysis patients (aged 21–65 years) without prior history of rabies vaccination during the last 20 years or significant immunosuppressive conditions | | | 0, 3, 7, 14, 28, 90 (2-2-2-0-1-1) ID | 0.1 mL ID  *PVRV 5 IU/ampule potency* | | | Group A (n=14)  GMTs, IU/mL (±SD)[range]/seroconversion rate   - Day 14: 3.2±3.1 [0.81–9.17]/100% - Day 90: 5.09±1.79 [0.42–25.0]/93% | |
| Sudarshan et al 2012 [20] | | India | | RFFIT | | | RCT | | | | Healthy adult volunteers (rabies vaccination history not stated; none had detectable RVNA) | | | Group A: 0, 3, 7 (4-4-4) ID (PVRV)  Group B: 0, 3, 7 (4-4-4) ID (PCECV)  Those with RVNA response (< 0.5 IU per mL) on day 365 were given ID booster (0,[4]) | Group A, 0.1 mL ID  Group B, 0.1 mL ID  *PVRV ≥2.5 IU/IM dose potency*  *PCECV ≥2.5 IU/IM dose potency* | | | All subjects  Group A (n=40)  GMTs, IU/mL (95% CI) [range]/seroconversion rate   - Day 7: 0.274 (0.241–0.311)[0.2–0.5]/20% - Day 14: 12.212 (11.693–12.755)[8.5–15.8]/100% - Day 28: 11.462 (10.925–12.025)[8.5–16.4]/100% - Day 180: 4.321 (4.102–4.551)[3.5–5.8]/100% - Day 365: 0.746 (0.628–0.886) [<0.5–1.5]/62.5%   Group B (n=38)  GMTs, IU/mL (95% CI) [range]/seroconversion rate   - Day 7: 0.266 (0.236–0.299)[0.2–0.5]/10.5% - Day 14: 12.010 (11.240–12.609)[8.5–14.7]/100% - Day 28: 11.296 (10.823–12.789)[8.5–15.6]/100% - Day 180: 4.325 (4.056–4.611)[2.5–6.5]/100% - Day 365: 0.717 (0.607–0.846) [<0.5–1.5]/78.9%   Booster for subjects with RVNA < 0.5 IU per mL on day 365  Group A (n=14)  GMTs, IU/mL (95% CI) [range]/seroconversion rate   - Day 7: 3.60 (3.11–4.17)[2.5–5.7]/100% - Day 14: 8.62 (7.81–9.51)[6.5–10.6]/100%   Group B (n=8)  GMTs, IU/mL (95% CI) [range]/seroconversion rate   - Day 7: 3.81 (3.13–4.64)[2.5–5.5]/100% - Day 14: 8.93 (7.92–10.08)[7.5–10.5]/100% | |
| Tantawichien et al 2014 [21] | | Thailand | | RFFIT | | | RCT | | | | Healthy adults aged ≥18 years (without a prior history of rabies vaccination or rabies seropositive) | | | Group A (PVRV): 0, 3, 7,14, 28 (2-2-2-0-2) ID: ID booster days 365 and 368 (0,3 [1-1]) after PEP  Group B (CPRV): 0, 3, 7, 14, 28 (2-2-2-0-2) ID: ID booster days 365 and 368 (0,3 [1-1]) after PEP  Group C (CPRV-TCRS): 0, 3, 7, 14, 28 (2-2-2-0-2) ID: ID booster days 365 and 368 (0,3 [1-1]) after PEP  All groups received concurrent purified ERIG: | Group A, 0.1 mL (0.1 mL booster)  Group B, 0.1 mL (0.1 mL booster)  Group C, 0.1 mL(0.1 mL booster)  *PVRV 10.6/ 18.6 booster IU/vial potency*  *CPRV 6.4/6.5 IU/vial potency*  *CPRV-TCRS 6.4 IU/vial potency* | | | Group A (n=31)  GMTs, IU/mL (95% CI) [range]/seroconversion rate   - Day 0: 0/0% - Day 14: 4.36 (2.66–5.36)[0.62–19.87]/100% - Day 28: 2.67 (1.49–3.61)[0.40–16.00]/90.3% - Day 90: 1.29 (0.89–1.89)[0.35–7.34]/90.3% - Day 180: 0.57 (0.41–0.82)[0.19–3.08]/51.7% - Day 365: 0.42 (0.26–0.66)[0.10–3.22]/50% - Day 379 (D14 booster): 15.12 (9.76–23.14)[2.38–412.29]/100%   Group B (n=35)  GMTs, IU/mL (95% CI) [range]/ seroconversion rate   - Day 0: 0/0% - Day 14: 9.98 (6.58–13.72)[1.14–69.79]/100% - Day 28: 6.95 (4.56–9.47)[0.74–49.35]/100% - Day 90: 2.84 (2.15–3.35)[1.09–20.75]/100% - Day 180: 1.31 (1.07–1.65)[0.32–9.51]/94.3% - Day 365: 0.92 (0.67–1.27)[0.16–7.34]/82.3% - Day 379 (D14 booster): 19.27 (14.18–26.19)[3.51–112.40]/100%   Group C (n=33)  GMTs, IU/mL (95% CI) [range]/seroconversion rate   - Day 0: 0/0% - Day 14: 7.70 (5.83–10.33)[0.96–29.54]/100% - Day 28: 4.63 (3.62–6.00)[0.59–14.67]/100% - Day 90: 2.90 (2.00–3.21)[0.81–16.0]/100% - Day 180: 0.96 (0.74–1.25)[0.34–4.56]/75.8% - Day 365: 0.78 (0.62–1.24)[0.23–4.0]/68.8% - Day 379 (D14 booster): 21.13 (15.64–28.54)[5.91–291.53]/100% | |
| Narayana et al 2015 [22] | | India | | RFFIT | | | RCT | | | | Adults aged 18–55 years with category II or category III exposures to rabies (with no previous history of rabies vaccination) | | | Group A: 0, 3, 7 (4-4-4) ID (PVRV)  Group B: 0, 3, 7 (4-4-4) ID (PCECV)  ERIG administered to all category III exposures (40 IU/kg body weight) | Group A, 0.1 mL ID  Group A, 0.1 mL ID  *PVRV 7.0 IU/dose potency*  *PCECV 7.5 IU/dose potency* | | | Group A (n=44)  GMCs, IU/mL (95% CI) [range]/seroconversion rate   - Day 14: 14.43 (13.41–15.53)[4.5–16.5]/100% - Day 90: 11.93 (11.47–12.40)[10.5–14.5]/100% - Day 365: 5.67 (5.29–6.08)[4.5–7.5]/100%   Group B (n=45)  GMCs, IU/mL (95% CI) [range]/seroconversion rate   - Day 14: 14.50 (13.50–15.57)[4.5–16.5]/100% - Day 90: 11.78 (11.27–12.31)[6.5–14.5]/100% - Day 365: 5.95 (5.50–6.44)[4.5–8.5]/100%   Data presented Cat II/III combined (i.e.. with and without ERIG combined data) | |
| Quiambao et al 2019 [23] | | Philippines | | RFFIT | | | RCT | | | | Participants aged <50 years (range 0–49 years) exposed to suspected rabid animals and sustaining WHO Category II injuries (automatic allocation to G1) or Category III injuries (randomized to G2 or G3) | | | Group A (Category II exposure): 0, 3, 7, 14, 28 (4-4-4-0-0) ID  Group B (Category III exposure): 0, 3, 7, 14, 28 (4-4-4-0-0) ID + ERIG (40 IU/kg)  Group C (Category III exposure): 0, 3, 7, 14, 28 (2-2-2-0-2) ID + ERIG (40 IU/kg) | Group A, 0.1 mL ID  Group B, , 0.1 mL ID  Group C, 0. 5 mL IM  *PVRV 7.7 and 9.1 IU/dose potency* | | | Group A (n=182)  GMTs, IU/mL (95% CI) [range]/seroconversion   - Day 14: 11.30 (9.94–12.9)[0.55–246]/100% - Day 90: 3.37 (2.97–3.82)[0.10–146]/98.4% - Day 360: 2.96 (2.57–3.40)[0.10–34.4]/97.6%   Group B (n=170)  GMTs, IU/mL (95% CI) [range]/seroconversion   - Day 14: 9.89 (8.36–11.7)[0.45–282]/99.4% - Day 90: 1.82 (1.60–2.07)[0.10–160]/94.3% - Day 360: 1.37 (1.20–1.58)[0.10–97.2]/89.0%   Group C (n=177)  GMTs, IU/mL (95% CI) [range]/seroconversion   - Day 14: 6.15 (5.26–7.20)[0.35–461]/98.9% - Day 90: 2.63 (2.37–2.92)[0.39–40.7]/98.4% - Day 360: 0.97 (0.85–1.10)[0.10–24.2]/79.8% | |
| Quiambao et al 2019 [23] up to 5 years + booster [24] | |  | |  | | |  | | | |  | | | 0 (4) ID booster |  | | | Group A (n=175)  GMTs, IU/mL (95% CI) /seroconversion   - Year 2: 3.29 (2.87–3.77)/98% - Year 3: 2.63 (2.29–3.02)/95.7% - Year 4: 2.27 (2.02–2.77)/96.2% - Year 5: 2.22 (1.90–2.60)/97.6%   Post-booster   - Day 11: 193(170–218)/100%   Group B (n=162)  GMTs, IU/mL (95% CI) /seroconversion   - Year 2: 1.47 (1.28–1.70)/88.5% - Year 3: 1.28 (1.12–1.47)/89.4% - Year 4: 0.918 (0.794–1.06)/80.1% - Year 5: 1.05 (0.918–1.21)/84.8%   Post-booster   - Day 11: 137 (120–157)/100%   Group B (n=162)  GMTs, IU/mL (95% CI) /seroconversion   - Year 2: 0.997 (0.863–1.15)/79.1% - Year 3: 0.863 (0.747–0.997)/71.8% - Year 4: 0.670 (0.559–0.804)/60.0% - Year 5: 0.762 (0.649–0.896)/64.1%   Post-booster   - Day 11: 138 (120–160)/100% | |
| Cantaert et al 2019 [25] | | Cambodia | | RFFIT | | | Observational | | | | Self-referred to patients (aged 7–39 years) bitten by a dog, had the biting dog’s head (for rabies antigen detection to establish exposure status), with no previous vaccination against rabies. | | | 0,3,7,28 (2-2-2-2)  Those with confirmed rabies exposure received purified ERIG (40 IU/kg) | 0.1 mL ID  *PVRV potency not stated* | | | Individuals (n=116) bitten by rabies virus-positive dogs. All had sustained category III exposure.  Median titers, IU/mL (IQR)   - Day 7: 1.08 (0.37–3.09) - Day 28: 26.86 (22.68–49.50) - Day 42: 26.74 (11.78–49.06)   Individuals (n=20) bitten by rabies virus-negative dogs  Median titers, IU/mL (IQR)   - Day 7: 2.54 (0.99–3.64) - Day 28: 49.5 (22.86–54.5) - Day 42: 26.86 (26.86–54.5)   Seroconversion achieved in 66% (90/136) at Day 7, and 100% by day 28 | |
| RAC17 (unpublished) [26] | | Philippines | | RFFIT | | | RCT | | | | Subjects aged > 2 years with a WHO Category I, II or III rabies exposure (without history of previous rabies immunization) | | | Group A: 0,3,7,14,28,90 (2-2-2-0-1-1)  Group B: 0,3,7,14,28,90 (2-2-2-0-2-0)  All the subjects received ERIG (40 IU/kg) | Group A, 0.1 mL ID  Group B, 0.1 mL ID  *PVRV ≥2.5 IU/0.5 mL dose potency* | | | Group A (n=48)  GMTs, IU/mL (95% CI) [range]/seroconversion   - Day 0: 0.028 (0.024–0.032)[0.02–0.180]/0% - Day 14: 80.8 (75.8–86.1)[39.0–113]/100% - Day 28: 43.8 (36.1–53.0)[8.70–93.0]/100% - Day 90: 5.42 (4.19–7.01)[0.88–35.0]/100%   Day 180: 4.29 (3.16–5.83)[0.36–34.0]/97.6%  Group B (n=82)  GMTs, IU/mL (95% CI) [range]/seroconversion   - Day 0: 0.026 (0.023–0.030)[0.02–0.44]/0% - Day 14: 72.9 (67.4–78.8)[17.0–113]/100% - Day 28: 45.4 (39.5–52.1)[8.3–113.0]/100% - Day 90: 6.68 (5.47–8.17)[1.10–84.0]/100% - Day 180: 3.33 (2.64–4.19)[0.33–57.0]/95.8% | |

Data shown for days since first dose

Data shown for PVRV unless otherwise stated

Seroconversion ≥0.5 IU/ml (protective level) unless specified

^#^1mL stated in publication but PVRV standard vial volume is 0.5mL (1 IM dose=1 vial of vaccine)

CPRV, chromatography purified Vero cell rabies vaccine; FAVN, fluorescent antibody virus neutralization; ID, intradermal; IM, intramuscular; MNT, mouse neutralization test; PCECV, purified chick embryo–cell rabies vaccine; PVRV, purified Vero cell rabies vaccine; RCT, randomized controlled study; RFFIT, rapid fluorescent focus inhibition test

**References**

1. Phanuphak, P.; Khawplod, P.; Sirivichayakul, S.; Siriprasomsub, W.; Ubol, S.; Thaweepathomwat, M. Humoral and cell-mediated immune responses to various economical regimens of purified Vero cell rabies vaccine. *Asian Pac J Allergy Immunol* **1987**, *5*, 33-37.

2. Chutivongse, S.; Wilde, H.; Supich, C.; Baer, G.M.; Fishbein, D.B. Postexposure prophylaxis for rabies with antiserum and intradermal vaccination. *Lancet* **1990**, *335*, 896-898, doi:10.1016/0140-6736(90)90488-q.

3. Phanuphak, P.; Khaoplod, P.; Benjavongkulchai, M.; Chutivongse, S.; Wilde, H. What happens if intradermal injections of rabies vaccine are partially or entirely injected subcutaneously? *Bull World Health Organ* **1990**, *68*, 83-85.

4. Kositprapa, C.; Limsuwun, K.; Wilde, H.; Jaijaroensup, W.; Saikasem, A.; Khawplod, P.; Kri-aksorn, U.; Supich, C. Immune response to simulated postexposure rabies booster vaccinations in volunteers who received preexposure vaccinations. *Clin Infect Dis* **1997**, *25*, 614-616, doi:10.1086/513760.

5. Tantawichien, T.; Benjavongkulchai, M.; Limsuwan, K.; Khawplod, P.; Kaewchompoo, W.; Chomchey, P.; Sitprija, V. Antibody response after a four-site intradermal booster vaccination with cell-culture rabies vaccine. *Clin Infect Dis* **1999**, *28*, 1100-1103, doi:10.1086/514737.

6. Briggs, D.J.; Banzhoff, A.; Nicolay, U.; Sirikwin, S.; Dumavibhat, B.; Tongswas, S.; Wasi, C. Antibody response of patients after postexposure rabies vaccination with small intradermal doses of purified chick embryo cell vaccine or purified Vero cell rabies vaccine. *Bull World Health Organ* **2000**, *78*, 693-698.

7. Tantawichien, T.; Tantawichien, T.; Supit, C.; Khawplod, P.; Sitprija, V. Three-year experience with 4-site intradermal booster vaccination with rabies vaccine for postexposure prophylaxis. *Clin Infect Dis* **2001**, *33*, 2085-2087, doi:10.1086/338155.

8. Tantawichien, T.; Jaijaroensup, W.; Khawplod, P.; Sitprija, V. Failure of multiple-site intradermal postexposure rabies vaccination in patients with human immunodeficiency virus with low CD4+ T lymphocyte counts. *Clin Infect Dis* **2001**, *33*, E122-124, doi:10.1086/324087.

9. Khawplod, P.; Wilde, H.; Tepsumethanon, S.; Limusanno, S.; Tantawichien, T.; Chomchey, P.; Ayuthaya, A.B.; Wangroonsarb, Y. Prospective immunogenicity study of multiple intradermal injections of rabies vaccine in an effort to obtain an early immune response without the use of immunoglobulin. *Clin Infect Dis* **2002**, *35*, 1562-1565, doi:10.1086/344954.

10. Madhusudana, S.N.; Sanjay, T.V.; Mahendra, B.J.; Suja, M.S. Simulated post-exposure rabies vaccination with purified chick embryo cell vaccine using a modified Thai Red Cross regimen. *Int J Infect Dis* **2004**, *8*, 175-179, doi:10.1016/j.ijid.2003.07.001.

11. Ambrozaitis, A.; Laiskonis, A.; Balciuniene, L.; Banzhoff, A.; Malerczyk, C. Rabies post-exposure prophylaxis vaccination with purified chick embryo cell vaccine (PCECV) and purified Vero cell rabies vaccine (PVRV) in a four-site intradermal schedule (4-0-2-0-1-1): an immunogenic, cost-effective and practical regimen. *Vaccine* **2006**, *24*, 4116-4121, doi:10.1016/j.vaccine.2006.02.036.

12. Khawplod, P.; Wilde, H.; Sirikwin, S.; Benjawongkulchai, M.; Limusanno, S.; Jaijaroensab, W.; Chiraguna, N.; Supich, C.; Wangroongsarb, Y.; Sitprija, V. Revision of the Thai Red Cross intradermal rabies post-exposure regimen by eliminating the 90-day booster injection. *Vaccine* **2006**, *24*, 3084-3086, doi:10.1016/j.vaccine.2006.01.051.

13. Madhusudana, S.N.; Sanjay, T.V.; Mahendra, B.J.; Sudarshan, M.K.; Narayana, D.H.; Giri, A.; Muhamuda, K.; Ravi, V.; Vakil, H.B.; Malerczyk, C. Comparison of saftey and immunogenicity of purified chick embryo cell rabies vaccine (PCECV) and purified vero cell rabies vaccine (PVRV) using the Thai Red Cross intradermal regimen at a dose of 0.1 ML. *Hum Vaccin* **2006**, *2*, 200-204, doi:10.4161/hv.2.5.3197.

14. Khawplod, P.; Wilde, H.; Benjavongkulchai, M.; Sriaroon, C.; Chomchey, P. Immunogenicity study of abbreviated rabies preexposure vaccination schedules. *J Travel Med* **2007**, *14*, 173-176, doi:10.1111/j.1708-8305.2007.00120.x.

15. Quiambao, B.P.; Gepanayao, C.; Bermal, N.; Ambas, M.C.; H., D.-T.; Crisostomo, M.; Dizon, R. Rabies vaccination regimens using purified Vero cell rabies vaccine. *APCRI Journal* **2008**, *X*.

16. Warrell, M.J.; Riddell, A.; Yu, L.M.; Phipps, J.; Diggle, L.; Bourhy, H.; Deeks, J.J.; Fooks, A.R.; Audry, L.; Brookes, S.M., et al. A simplified 4-site economical intradermal post-exposure rabies vaccine regimen: a randomised controlled comparison with standard methods. *PLoS Negl Trop Dis* **2008**, *2*, e224, doi:10.1371/journal.pntd.0000224.

17. Sampath, G.; Madhusudana, S.N.; Sudarshan, M.K.; Ashwathnarayana, D.H.; Mahendra, B.J.; Ullas, T.P.; Mohan, K.; Madhusudhan, S.K.; Ravish, H.S. Immunogenicity and safety study of Indirab: a Vero cell based chromatographically purified human rabies vaccine. *Vaccine* **2010**, *28*, 4086-4090, doi:10.1016/j.vaccine.2010.03.064.

18. Shantavasinkul, P.; Tantawichien, T.; Wilde, H.; Sawangvaree, A.; Kumchat, A.; Ruksaket, N.; Lohsoonthorn, V.; Khawplod, P.; Tantawichien, T. Postexposure rabies prophylaxis completed in 1 week: preliminary study. *Clin Infect Dis* **2010**, *50*, 56-60, doi:10.1086/649211.

19. Tanisaro, T.; Tantawichien, T.; Tiranathanagul, K.; Susantitaphong, P.; Chirananthavat, T.; Praditpornsilpa, K.; Sitprija, V.; Eiam-Ong, S. Neutralizing antibody response after intradermal rabies vaccination in hemodialysis patients. *Vaccine* **2010**, *28*, 2385-2387, doi:10.1016/j.vaccine.2010.01.003.

20. Sudarshan, M.K.; Narayana, D.H.; Madhusudana, S.N.; Holla, R.; Ashwin, B.Y.; Gangaboraiah, B.; Ravish, H.S. Evaluation of a one week intradermal regimen for rabies post-exposure prophylaxis: results of a randomized, open label, active-controlled trial in healthy adult volunteers in India. *Hum Vaccin Immunother* **2012**, *8*, 1077-1081, doi:10.4161/hv.20471.

21. Tantawichien, T.; Sibunruang, S.; Tantawichien, T.; Angsanakul, J.; Benjavongkulchai, M.; Limsuwan, K.; Udomchaisakul, P.; Khomvilai, S.; Sitprija, V. Safety and immunogenicity of chromatographically purified Vero cell rabies vaccine for intradermal pre- and post-exposure rabies prophylaxis. *Expert Rev Vaccines* **2014**, *13*, 1593-1601, doi:10.1586/14760584.2014.971764.

22. Narayana, A.; Manoharan, A.; Narayan, M.S.; Kalappa, S.M.; Biligumba, G.; Haradanahalli, R.; Anand, A.M. Comparison of safety and immunogenicity of 2 WHO prequalified rabies vaccines administered by one week, 4 site intra dermal regimen (4-4-4-0-0) in animal bite cases. *Hum Vaccin Immunother* **2015**, *11*, 1748-1753, doi:10.1080/21645515.2015.1048938.

23. Quiambao, B.P.; Ambas, C.; Diego, S.; Bosch Castells, V.; Korejwo, J.; Petit, C.; Houillon, G. Intradermal post-exposure rabies vaccination with purified Vero cell rabies vaccine: Comparison of a one-week, 4-site regimen versus updated Thai Red Cross regimen in a randomized non-inferiority trial in the Philippines. *Vaccine* **2019**, *37*, 2268-2277, doi:10.1016/j.vaccine.2019.02.083.

24. Quiambao, B.P.; Ambas, C.; Diego, S.; Bosch Castells, V.; Korejwo, J.; Petit, C.; Rasuli, A.; Houillon, G. A single-visit, 4-site intradermal (ID) rabies vaccination during simulated post-exposure induces a robust immune response 5 years after primary 1-week, 4-site ID post-exposure prophylaxis regimen in the Philippines. *Vaccine (Submitted 2019)* **2019**.

25. Cantaert, T.; Borand, L.; Kergoat, L.; Leng, C.; Ung, S.; In, S.; Peng, Y.; Phoeun, C.; Hing, C.; Taing, C.N., et al. A 1-week intradermal dose-sparing regimen for rabies post-exposure prophylaxis (RESIST-2): an observational cohort study. *Lancet Infect Dis* **2019**, 10.1016/S1473-3099(19)30311-1, doi:10.1016/S1473-3099(19)30311-1.

26. RAC17. Clinical Study Report (unpublished). Combined immunogenicity of Chromatographically Purified Rabies Vaccine (CPRV, with and without Merthiolate) and equine rabies immune globulin (ERIG), in comparison with Purified Vero Rabies Vaccine (PVRV) and ERIG, administered by intradermal route, in subjects with a WHO Category I, II, or III Rabies exposure. 13 March 2002.
