# Supplementary material for: Purified Vero Cell Rabies Vaccine (PVRV, Verorab®): A Systematic Review of Intradermal Use Between 1985 and 2019
Source: Trop Med Infect Dis. 2020 Mar 7;5(1):40. doi: 10.3390/tropicalmed5010040 (PMC7157209; doi:10.3390/tropicalmed5010040)

Figure S1: Summary of post-exposure prophylaxis (PEP) immunogenicity data from all studies irrespective of regimen [regimen summarized as day of vaccination (number of sites per vaccination day) in legend key]. Dashed line marks the 0.5IU/mL RVNA titer.

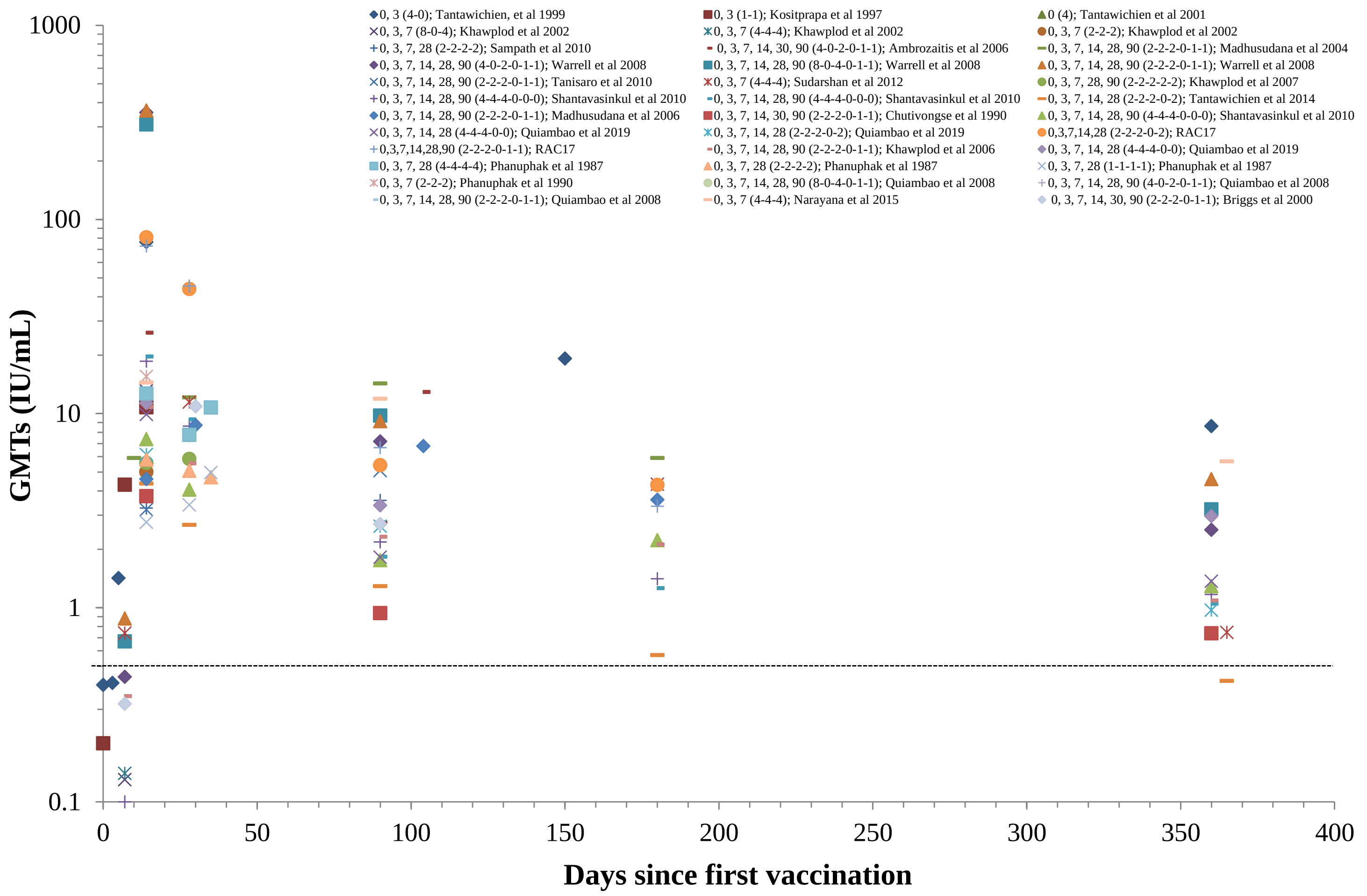

Supplement: Supplementary file 1 [file tropicalmed-05-00040-s001.zip › Figure S1.pdf]
